# Nuclear Translocation and Regulation of Intranuclear Distribution of Cytoplasmic Poly(A)-Binding Protein Are Distinct Processes Mediated by Two Epstein Barr Virus Proteins

**DOI:** 10.1371/journal.pone.0092593

**Published:** 2014-04-04

**Authors:** Richard Park, Ayman El-Guindy, Lee Heston, Su-Fang Lin, Kuan-Ping Yu, Mate Nagy, Sumit Borah, Henri-Jacques Delecluse, Joan Steitz, George Miller

**Affiliations:** 1 Department of Molecular Biophysics and Biochemistry, Yale University School of Medicine, New Haven, Connecticut, United States of America; 2 Department of Pediatrics, Yale University School of Medicine, New Haven, Connecticut, United States of America; 3 Institute of Cancer Research, National Health Research Institutes, Zhunan Town, Taiwan; 4 Program in Computational Biology and Bioinformatics, Yale University, New Haven, Connecticut, United States of America; 5 Department of Biochemistry, Howard Hughes Medical Institute, University of Colorado Biofrontiers Institute, Boulder, Colorado, United States of America; 6 Department of Tumor Virology, German Cancer Research Center, Heidelberg, Germany; 7 Department of Epidemiology and Public Health, Yale University School of Medicine, New Haven, Connecticut, United States of America; University of Sussex, United Kingdom

## Abstract

Many viruses target cytoplasmic polyA binding protein (PABPC) to effect widespread inhibition of host gene expression, a process termed viral host-shutoff (vhs). During lytic replication of Epstein Barr Virus (EBV) we observed that PABPC was efficiently translocated from the cytoplasm to the nucleus. Translocated PABPC was diffusely distributed but was excluded from viral replication compartments. Vhs during EBV infection is regulated by the viral alkaline nuclease, BGLF5. Transfection of BGLF5 alone into BGLF5-KO cells or uninfected 293 cells promoted translocation of PAPBC that was distributed in clumps in the nucleus. ZEBRA, a viral bZIP protein, performs essential functions in the lytic program of EBV, including activation or repression of downstream viral genes. ZEBRA is also an essential replication protein that binds to viral oriLyt and interacts with other viral replication proteins. We report that ZEBRA also functions as a regulator of vhs. ZEBRA translocated PABPC to the nucleus, controlled the intranuclear distribution of PABPC, and caused global shutoff of host gene expression. Transfection of ZEBRA alone into 293 cells caused nuclear translocation of PABPC in the majority of cells in which ZEBRA was expressed. Co-transfection of ZEBRA with BGLF5 into BGLF5-KO cells or uninfected 293 cells rescued the diffuse intranuclear pattern of PABPC seen during lytic replication. ZEBRA mutants defective for DNA-binding were capable of regulating the intranuclear distribution of PABPC, and caused PABPC to co-localize with ZEBRA. One ZEBRA mutant, Z(S186E), was deficient in translocation yet was capable of altering the intranuclear distribution of PABPC. Therefore ZEBRA-mediated nuclear translocation of PABPC and regulation of intranuclear PABPC distribution are distinct events. Using a click chemistry-based assay for new protein synthesis, we show that ZEBRA and BGLF5 each function as viral host shutoff factors.

## Introduction

Viruses promote a widespread reduction of host cell gene expression to reduce competition for cellular resources, to decrease expression of cellular factors that elicit an immune response to viral infection, and to facilitate the establishment of viral latency. This process, termed viral host shutoff (vhs), is mediated by modulation of transcription, mRNA splicing, nuclear export of mRNA, mRNA decay, translation, and proteolysis [1]. Cytoplasmic polyadenylate binding protein C, (PABPC), a regulator of mRNA stability and a contributor to translation initiation, is targeted by many viruses. Several classes of RNA viruses, including picornaviruses [2]–[4], caliciviruses [4] and lentiviruses [5] hinder translation of host mRNA by proteolytic cleavage of PABPC by virally encoded proteases. Rotaviruses do not cleave PABPC, but they inhibit PABPC-mediated cap-dependent translation initiation. NSP3 (non-structural protein 3) evicts PABPC from eukaryotic mRNA poly(A) tails and disrupts the interaction between PABPC and eIF4G [6], [7]. PABPC accumulates in the nucleus as the result of an interaction of NSP3 with a cellular protein, RoXaN [8], [9].

Among herpesviruses, the alphaherpesvirus herpes simplex virus type 1 (HSV-1), and the gammaherpesviruses Kaposi's sarcoma-associated herpesvirus (KSHV), murine gammaherpesvirus 68 (MHV68), and Epstein-Barr virus (EBV), all induce vhs characterized by accelerated global host mRNA decay during the lytic phases of replication. Betaherpesviruses, like human cytomegalovirus (HCMV), in contrast, do not shut-off host macromolecular synthesis [10]. Relocalization of PABPC from the cytoplasm to the nucleus is a component of the host-shutoff by alphaherpesviruses and gammaherpesviruses, but the mechanisms and viral factors mediating host-shutoff differ. Host-shutoff induced by HSV-1 is regulated primarily by the *vhs* protein, an endonuclease with sequence homology to the FEN-1 family of nucleases, which rapidly degrades mRNAs [11]. During lytic HSV-1 infection, translocation of PABPC is mediated by *vhs* [12] and a second viral protein, ICP27, that interacts directly with PABPC and promotes nuclear translocation of PABPC in the absence of other viral factors [13]. Infection with an ICP27-null mutant HSV-1 also results in nuclear translocation of PABPC; redundant viral or cellular factors may mediate the translocation of PABPC during HSV-1 infection [14].

During lytic infection by KSHV, vhs and translocation of PABPC is mediated by SOX (**S**hut**O**ff and e**X**onuclease), a viral alkaline nuclease (AN) encoded by ORF37, a gene that is conserved among all herpesvirus family members [15], [16]. SOX was identified as the sole mediator of the host shutoff in a screen of 76 KSHV genes assessing downregulation of a reporter, green fluorescent protein [15]. SOX was sufficient to induce global host mRNA turnover and translocation of PABPC to the nucleus in the absence of other viral factors. Endonucleolytic cleavage of mRNAs by SOX recruits the host Xrn1 exonuclease, which degrades mRNAs leading to importin-α-mediated translocation of released PABPC into the nucleus [17]. Accumulation of intranuclear PABPC causes excessive hyperadenylation of nuclear mRNAs and a block to export of hyperadenylated mRNAs from the nucleus [12]. In KSHV infected cells activated into the lytic cycle and in uninfected cells transfected with SOX, translocated PABPC distributes diffusely throughout the nucleus and co-localizes with hyperadenylated mRNAs and with SOX [12], [16], [17].

A proposed model postulates that, by binding to extended poly(A)-tails and by sequestering hyperadenylated mRNAs in the nucleus, intranuclear PABPC precludes translation of cellular mRNAs [12]. The importance of the translocation of PABPC itself to inhibition of gene expression was demonstrated by fusing PABPC to a nuclear retention signal (Flag-PABPC1-NRS). In the absence of SOX or other viral factors, Flag-PABPC1-NRS caused a rapid increase in retention of poly(A)-mRNAs in the nucleus [12]. In experiments with a GFP reporter, Flag-PABPC1-NRS caused an increase in hyperadenylated GFP mRNA, a decrease in normally polyadenylated GFP mRNA, and a decrease in levels of GFP protein [12].

After SOX was shown to be the primary inducer of vhs by KSHV, the AN homologs in EBV (BGLF5) and MHV68 (muSOX) were also found to induce host shutoff and to translocate PABPC from the nucleus to the cytoplasm when transiently transfected into cells lacking virus [16], [18]–[20]. However, it has not been investigated whether PABPC undergoes relocalization during lytic infection of EBV, whether EBV factors in addition to BGLF5 regulate nuclear accumulation of PABPC, and whether additional viral factors contribute to vhs during lytic induction of EBV.

In this study, we examined in detail the nuclear translocation of PABPC during the early stages of lytic EBV infection. We report that in addition to BGLF5, the major lytic cycle regulatory protein, ZEBRA, controls the intracellular localization of PABPC and regulates host shutoff during lytic infection. ZEBRA is a member of the bZIP family of transcription factors, and is expressed from the BZLF1 gene as an early lytic protein. As an essential transcription factor and replication protein, ZEBRA binds DNA at specific sequences termed ZEBRA response elements (ZRE), and activates or represses downstream lytic viral genes. In cells lacking the EBV genome, the combination of BGLF5 and ZEBRA were sufficient to re-locate PABPC in the nucleus in a pattern seen during lytic infection. ZEBRA and BGLF5 each individually elicited a distinct nuclear distribution pattern of PABPC; ZEBRA co-localized with intranuclear PABPC, whereas BGLF5 did not. While both ZEBRA and BGLF5 were capable of promoting PABPC accumulation in the nucleus, ZEBRA was dominant in influencing a diffuse intranuclear distribution of PABPC. We also show that both BGLF5 and ZEBRA function as regulators of host shutoff. Each protein caused a global inhibition of endogenous host protein synthesis.

## Results

### Cytoplasmic poly(A) binding protein (PABPC) translocates to the nucleus during the EBV lytic cycle

**Figure 1:**
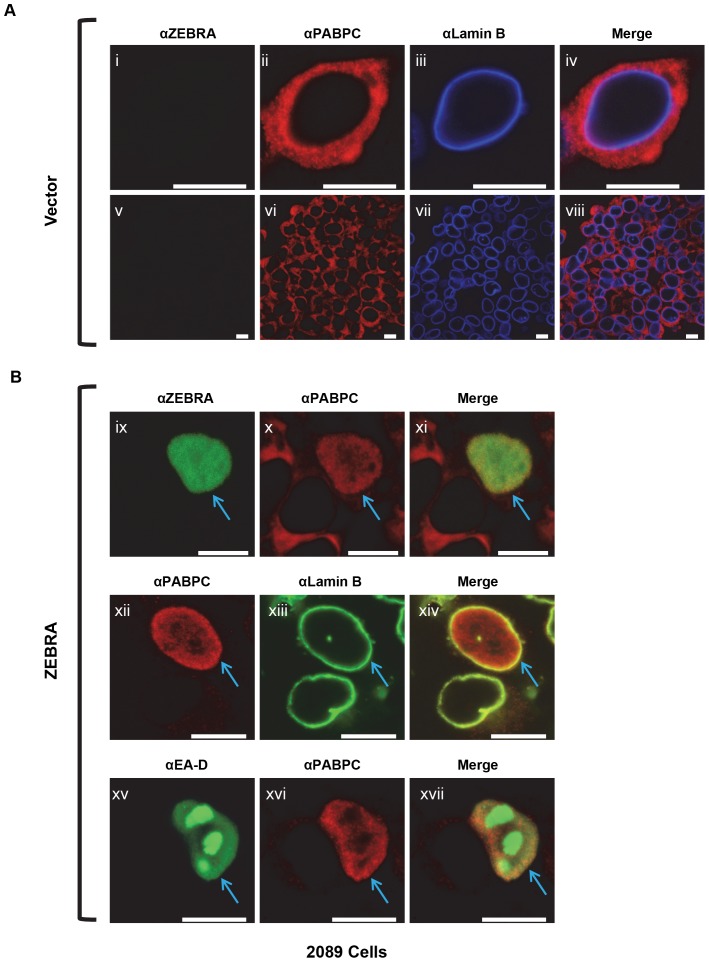
Induction of the lytic cycle in 293 cells containing an intact EBV-bacmid (2089 cells) is accompanied by translocation of PABPC to a diffuse distribution in the nucleus. 2089 cells were transfected with (A) vector (pHD1013), or (B) an expression vector for WT ZEBRA (pCMV-gZ). Cells were fixed and stained with antibodies specific for ZEBRA (green) (i, iv, v, viii, ix, xi), PABPC (red) (ii, iv, vi, viii, x, xi, xii, xiv,xvi,xvii), lamin B [iii, iv, vii, viii,(blue) xiii, xiv(green)], or EA-D(green) (xv, vii) and fluorophore-conjugated secondary antibodies. Digital images were acquired by confocal microscopy. Each of the following sets of panels depicts the same field of view: [i-iv], [v-vii], [viii-x], [xi-xiii]. Blue arrows denote cells in which PABPC localized to the interior of the nucleus. Reference bar in each panel equals 10 μM in length.

In preliminary experiments, the localization of PABPC was examined in HH514-16, a cell line derived from Burkitt lymphoma, untreated or treated with sodium butyrate to induce the EBV lytic cycle (Fig. S1). In untreated cells, PABPC was exclusively cytoplasmic (Fig. S1: iii). In lytically induced cells, PABPC was present in the nucleus in cells that were positive for diffuse early antigen (EA-D) a viral protein that functions as a DNA polymerase processivity factor during lytic replication (Fig. S1: v, vi). To investigate the cell biology and mechanism of PABPC translocation in more detail, we used 293 human embryonic kidney epithelial cells containing EBV bacmids [21]–[23]. These cells permit better visualization of subcellular localization and allow the use of EBV genetics to analyze the contribution of individual gene products to different phases of the EBV lytic cycle. For initial experiments we used 2089 cells, which carry a bacmid with an intact EBV genome. When 2089 cells were transfected with an empty vector (pHD1013), PABPC was located exclusively in the cytoplasm (Fig. 1A); this localization of PABPC was identical in cells that had not been transfected (not shown). When the EBV lytic cycle was induced by transfection of a plasmid expressing ZEBRA, PABPC localized to the nucleus (Fig. 1B: x, xi, xii, xiv, xvi, xvii; blue arrows). Co-staining of PABPC and lamin B showed that translocated PABPC was diffusely distributed throughout the nucleus (Fig. 1B: xii-xiv; blue arrows). Close observation of intranuclear PABPC showed it to have a finely speckled pattern, sparing small subnuclear regions and often concentrated at the nuclear periphery (Fig. 1B: xii, xvi). Immunoblot analysis of whole cell extracts showed that total PABPC levels remained relatively unchanged during lytic activation (Fig. S2).

### Nuclear translocation of PABPC occurs in the absence of replication compartments

The lytic cycle of EBV progresses through distinct temporal stages: the early stage is defined by expression of viral “early genes” many of which encode proteins required for DNA replication; early gene expression is followed by the onset of viral DNA replication in which viral DNA is synthesized in subnuclear globular domains called replication compartments; viral DNA replication permits entry into the late stage of lytic infection in which viral “late genes” are expressed and virions are produced. Lytically induced cells were co-stained with antibodies to PABPC and to EA-D (early antigen-diffuse), a viral gene product whose intranuclear distribution differs during the early and late phases of the EBV life cycle. EA-D is diffusely present throughout the nucleus during early phases of the life cycle and concentrates in replication compartments during and after DNA replication. Three hundred-forty-four cells expressing EA-D, chosen at random, were scored for the localization of EA-D and PAPBC (Table 1). PABPC was translocated to the nucleus of 74% of cells that expressed EA-D but did not contain replication compartments, a pattern characteristic of the early gene stage; 26% of early stage cells positive for EA-D did not show translocation of PABPC. PABPC was present in the nucleus of all cells with globular viral replication compartments indicating active viral DNA replication or subsequent lytic stages of infection. These results indicate that translocation of PABPC occurs before formation of replication compartments and is coincident with early viral gene expression. Co-staining with EA-D during the late replicative phase showed that PABPC that was translocated to the nucleus was excluded from globular replication compartments (Fig. 1B: xv-xvii).

**Table 1:** Translocation of PABPC to the nucleus occurs in cells induced into the EBV lytic cycle whether or not they contain visible replication compartments.

Total # of Cells Positive for EA-D: 344			
# Cells Containing Diffuse EA-D (No Replication Compartments): 281		# Cells Containing Globular EA-D (Replication Compartments): 63	
# Cells with PABPC Translocation:208 (74%)	# Cells with No PABPC Translocation:73 (26%)	# Cells with PABPC Translocation:63 (100%)	# Cells with No PABPC Translocation:0 (0%)
2089 Cells			

2089 cells were transfected with an expression vector for ZEBRA. The cells were fixed 40 hours after transfection and co-stained for the early EBV lytic gene product, EA-D and evaluated for the presence of PABPC in the nucleus.

### EBV BGLF5 mediates translocation of PABPC to the nucleus

**Figure 2:**
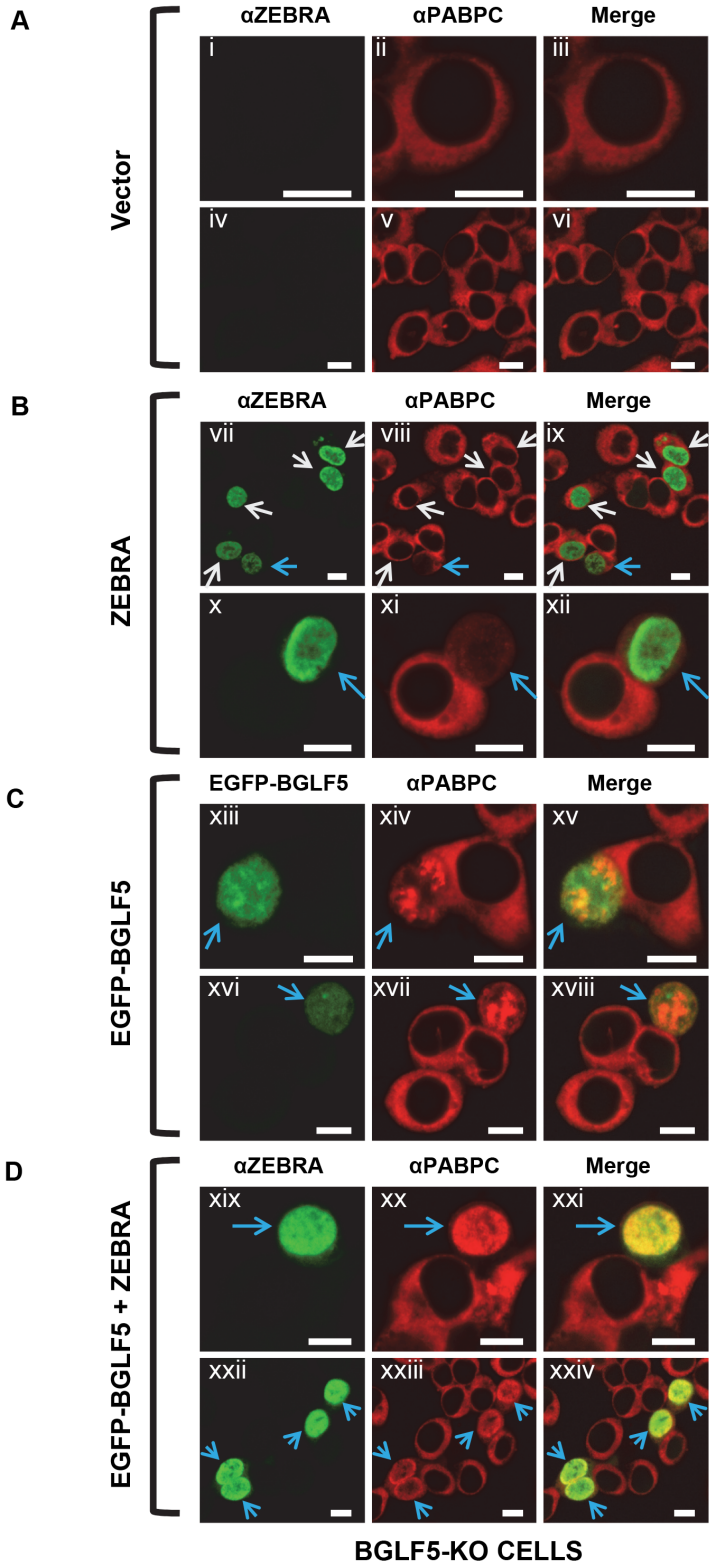
The EBV BGLF5 protein induces nuclear translocation of PABPC, but does not reproduce the diffuse sub-nuclear distribution of PABPC seen during lytic replication. BGLF5-KO cells were transfected with: (A) vector, (B) ZEBRA, (C) EGFP-BGLF5, or (D) ZEBRA and EGFP-BGLF5. Cells were fixed and stained with antibodies specific for ZEBRA and PABPC, and fluorophore-conjugated secondary antibodies. BGLF5 expression was indicated by EGFP. When EGFP-BGLF5 and ZEBRA were co-expressed, ZEBRA protein was detected at a PMT setting that was insufficient to detect EGFP. Each of the following sets of panels depicts the same field of view: [i-iii], [iv-vi], [vii-ix], [x-xii], [xiii-xv], [xvi-xviii], [xix-xxi], [xxii-xxiv]. White arrows in [vii-ix] denote cells expressing ZEBRA with no nuclear translocation of PABPC; blue arrows in [vii-ix], [x-xii], [xiii-xv], [xvi-xviii], [xix-xxi], and [xxii-xxiv] denote cells expressing ZEBRA or EGFP-BGLF5 and exhibiting translocation of PABPC to the nucleus. Reference bar in each panel equals 10 μM in length.

We asked whether BGLF5, the EBV homologue of KSHV SOX and MHV68 muSOX, functions similarly to translocate PABPC to the nucleus [16]. In these experiments we used a 293 cell line containing an EBV bacmid with insertional inactivation of the BGLF5 gene (BGLF5-KO) [23]. In BGLF5-KO cells containing latent EBV transfected with empty vector, PABPC was exclusively cytoplasmic (Fig. 2A). When BGLF5-KO cells were transfected with ZEBRA to induce the EBV lytic cycle, intranuclear PABPC was seen in a sub-population of cells that expressed ZEBRA (Fig. 2B; blue arrows). In these cells the nuclear PABPC staining was faint and some PABPC remained in the cytoplasm (Fig. 2B: viii, ix, xi, xii). These results show that while BGLF5 is necessary for maximal PABPC translocation, partial translocation or retention of PABPC in the nucleus occurs in the absence of BGLF5 and the presence of ZEBRA. PABPC was found in the nucleus (Fig. 2C) in BGLF5-KO cells transfected with a BGLF5 expression vector. However, the intranuclear distribution of PABPC following transfection of BGLF5 was uneven, clumped and aggregated (Fig. 2C: xiv, xvii; blue arrows). No cells with BGLF5 alone showed the diffuse distribution of intranuclear PABPC characteristic of lytic infection.

These results suggested that an EBV lytic cycle product other than BGLF5 regulates the intranuclear distribution of translocated PABPC characteristic of the lytic cycle. To test this hypothesis, BGLF5-KO cells were co-transfected with BGLF5 and with ZEBRA to induce the lytic cycle and thereby provide additional lytic cycle proteins (Fig. 2D). Under these conditions, PABPC was efficiently translocated to the nucleus, stained intensely and distributed diffusely in a pattern identical to that seen in lytically induced 2089 cells. These results suggest that although BGLF5 mediates nuclear translocation of PABPC, additional viral or cellular factors present during lytic infection control the intranuclear distribution of PABPC.

### BGLF5 and ZEBRA regulate translocation of PABPC and its distribution in the nucleus independent of other viral genes

**Figure 3:**
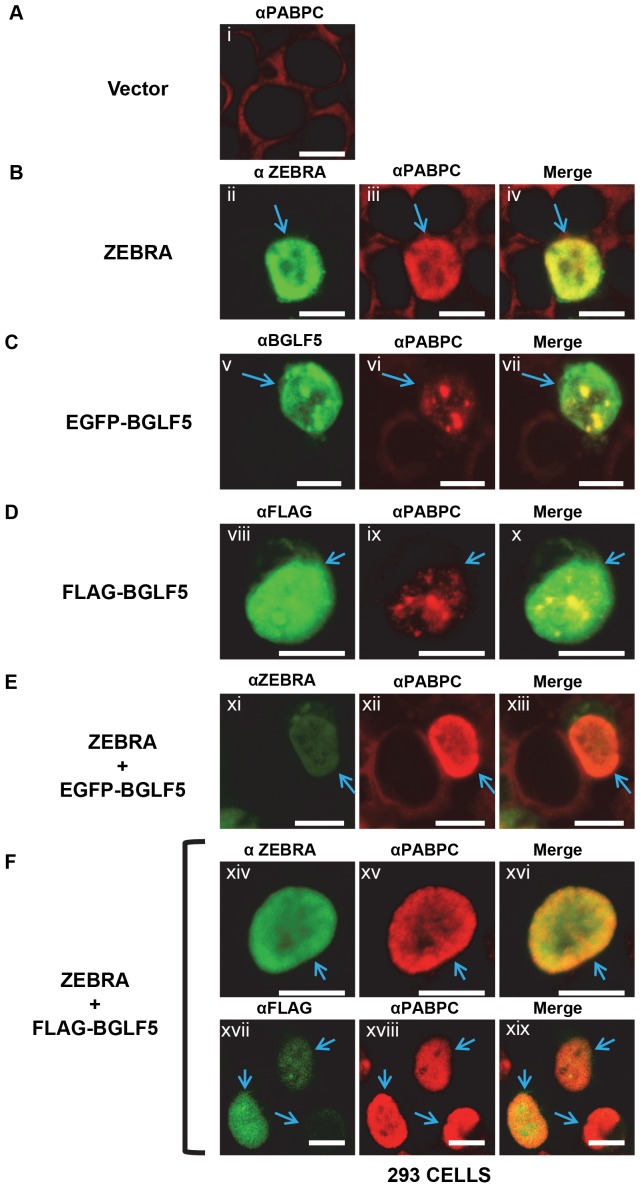
BGLF5 and ZEBRA independently regulate translocation of PABPC and its distribution in the nucleus. 293 cells were transfected with: (A) vector, (B) ZEBRA, (C) EGFP-BGLF5, (D) FLAG-BGLF5, (E) ZEBRA and EGFP-BGLF5, or (F) ZEBRA and FLAG-BGLF5. Cells were fixed and stained with antibodies specific for PABPC, FLAG, or ZEBRA, and fluorophore-conjugated secondary antibodies. Each of the following sets of panels depicts the same field of view: [ii-iv], [v-vii], [viii-x], [xi-xiii], [xiv-xvi], [xvii-xix]. Blue arrows indicate cells in which PABPC localized to the nucleus. Reference bar in each panel equals 10 μM in length.

Using 293 cells lacking EBV, we studied whether BGLF5 or ZEBRA could mediate nuclear translocation of PABPC in the absence of all other viral products. In 293 cells, PABPC remained exclusively cytoplasmic after transfection of an empty vector (Fig. 3A). Transfection of ZEBRA alone into 293 cells resulted in a mixed population of cells showing two phenotypes. In approximately one-third of cells expressing ZEBRA, PABPC was not present in the nucleus. Two-thirds of 293 cells transfected with ZEBRA showed intranuclear staining of PABPC (Fig. 3B: ii-iv: blue arrows). This result indicates that ZEBRA plays a partial role in mediating translocation of PABPC from the cytoplasm to the nucleus in the absence of other viral factors.

Transfection of BGLF5 expression vectors promoted nuclear translocation of PABPC in all 293 cells that expressed BGLF5 protein (Fig. 3C, 3D). The clumped intranuclear distribution of PABPC observed in 293 cells is indistinguishable from the pattern of distribution seen in BGLF5-KO cells transfected with the EGFP-BGLF5 expression vector (Fig. 2C). The same clumped intranuclear distribution of PABPC was observed when the BGLF5 expression vector was fused to EGFP (Fig. 3C: v-vii) or to FLAG (Fig. 3D: viii-x). When BGLF5 was co-transfected with ZEBRA into 293 cells (Fig. 3E, 3F), PABPC was translocated efficiently into the nucleus, and was diffusely distributed, similar to the pattern seen in lytically induced 2089 cells Fig. 1B) or in BGLF5-KO cells co-transfected with BGLF5 and ZEBRA (Fig. 2D). We conclude that ZEBRA promotes a diffuse distribution of PABPC in the nucleus.

To investigate the specificity of ZEBRA's effect on the localization of PABPC, we tested the ability of Rta, another EBV early viral transcription factor that localizes exclusively to the nucleus, to regulate the distribution of translocated PABPC [24], [25]. Rta functions in concert with ZEBRA to activate downstream lytic viral genes and to stimulate viral replication. Transfection of 293 cells with a Rta expression vector (pRTS-Rta) produced high levels of Rta protein; however, there was no translocation of PABPC to the nucleus in any cell (data not shown). To determine whether Rta could promote a diffuse distribution pattern of intranuclear PABPC, Rta was co-transfected with BGLF5 (Fig. S3). Under these conditions, PABPC was translocated but clumped in the nucleus (Fig. S3: ii, iii): the distribution of PABPC was the same in cells transfected with BGLF5 alone or BGLF5 plus Rta.

**Figure 4:**
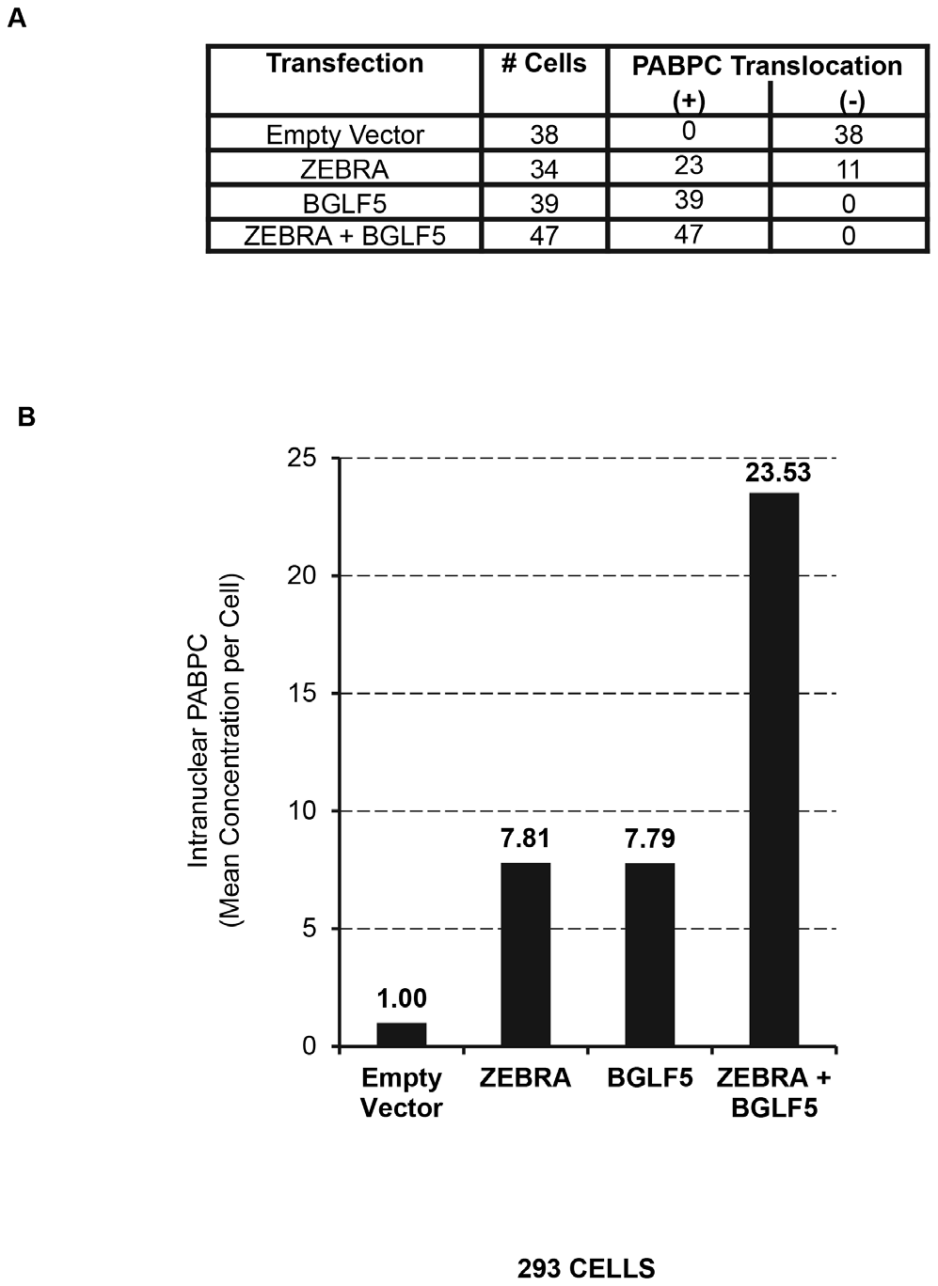
Frequency and intensity of PABPC-translocation induced by ZEBRA and BGLF5. 293 cells were transfected with vector, ZEBRA, or EGFP-BGLF5, or co-transfected with ZEBRA and EGFP-BGLF5. Cells were fixed and stained with antibodies specific for PABPC and ZEBRA, and fluorophore-conjugated secondary antibodies. Digital images were acquired by confocal microscopy and analyzed by ImageJ software (NIH). (A) Numbers of cells that were positive and negative for translocation of PABPC for each transfection condition. (B) Concentrations of intranuclear PABPC were measured by ImageJ software; 34 to 47 cells selected at random for each transfection condition. Measurements of intranuclear PABPC were normalized to the mean average value of 1.00 for the empty vector control.

Several aspects of the translocation of PABPC in 293 cells transfected with ZEBRA and BGLF5, individually or in combination, were quantitated (Fig. 4A). First, we scored the number of cells showing PABPC translocation. In cells transfected with ZEBRA alone, 23 of 34 randomly selected cells expressing ZEBRA showed translocation of PABPC. In contrast, in cells transfected with BGLF5 alone, 100% of 39 randomly selected cells expressing BGLF5 showed translocation of PABPC; likewise, 100% of 47 randomly selected cells expressing both ZEBRA and BGLF5 showed translocation of PABPC. Second, the extent of translocation of PABPC induced by ZEBRA or BGLF5 was quantified using ImageJ software analysis of the same transfected 293 cells (Fig. 4B). The mean average fluorescence signal of PABPC within nuclei of 38 cells transfected with the vector control was normalized to a value of 1.00 per cell. Measurement of translocated PABPC within each of the 23 cells positive for ZEBRA expression and for PABPC translocation showed a 7.81-fold mean increase of intranuclear PABPC per cell compared to the vector control. Measurement of PABPC translocation in the 39 cells transfected with BGLF5 alone showed a nearly identical mean average of 7.79 per cell. Measurement of PABPC translocation in cells co-transfected with ZEBRA and BGLF5 gave a mean average of 23.53 per cell. Taken together, these results showed that: i) whereas BGLF5 induced translocation of PABPC in every cell, ZEBRA induced translocation in a smaller proportion, approximately two-thirds, of cells; ii) on a single cell basis, however, the extent of translocation of PABPC induced by ZEBRA and BGLF5 were nearly the same; iii) co-transfection of ZEBRA and BGLF5 were synergistic in PABPC translocation. The amount of PABPC within a single nucleus of cells exposed to both proteins (ImageJ value of 23.53; 100%) was greater than the sum of single-cell PABPC translocations caused by ZEBRA alone (7.81; 33.2%) plus BGLF5 alone (7.79; 33.1%).

### ZEBRA controls the intranuclear distribution of PABPC

A FLAG-tagged version of PABPC aberrantly mis-localizes to the nucleus of uninfected 293 cells and distributes unevenly in clumps and aggregates (Fig. S4A). When FLAG-PABPC was co-transfected with ZEBRA (Fig. S4B), the clumped appearance of PABPC was replaced with an evenly diffuse distribution similar to that seen during lytic induction. Thus, ZEBRA alone causes the diffuse distribution of intranuclear PABPC, independent of BGLF5 expression. The specificity of ZEBRA in controlling the intranuclear distribution of PABPC was tested using another bZIP protein, the AP-1 transcription factor c-Jun. Co-transfection with c-Jun did not alter the clumped and aggregated distribution of FLAG-PABPC (Fig. S4C), indicating that control of the intranuclear distribution of PABPC is specific to ZEBRA.

### Both ZEBRA and translocated PABPC spare nucleoli

**Figure 5:**
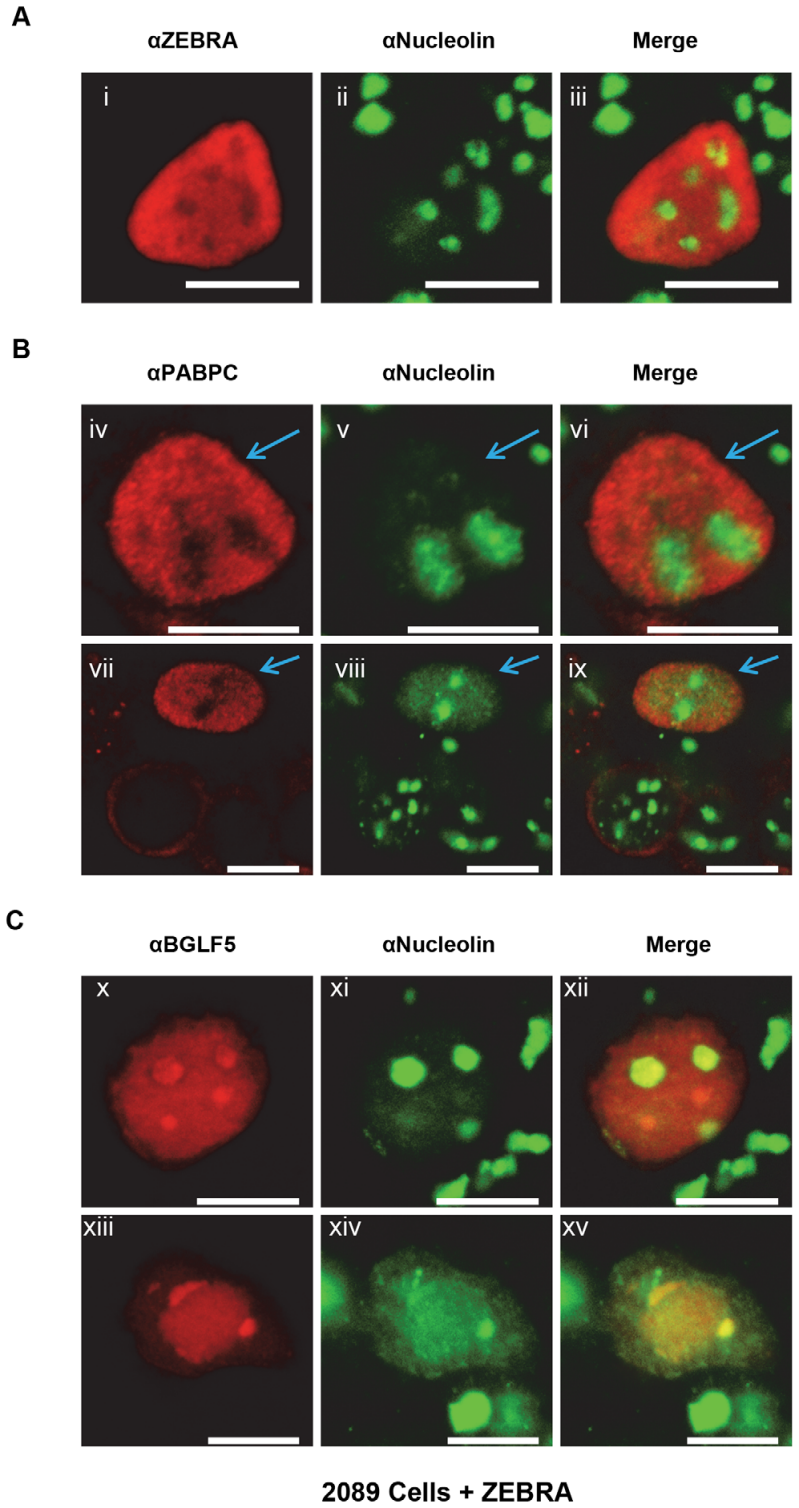
During the EBV lytic cycle, ZEBRA and translocated PABPC spare nucleoli, whereas BGLF5 is enriched in nucleoli. 2089 cells were transfected with ZEBRA to induce the lytic phase. Cells were fixed and stained with antibodies specific for ZEBRA, nucleolin, PABPC, or BGLF5, and fluorophore-conjugated secondary antibodies. Blue arrows in [iv-vi] and [vii-ix] indicate cells in which PABPC localized to the nucleus. Each of the following sets of panels depicts the same field of view: [i-iii], [iv-vi], [vii-ix], [x-xii], [xiii-xv]. Reference bar in each panel equals 10 μM in length.

During the EBV lytic phase, diffusely distributed intranuclear PABPC was often concentrated at the nuclear periphery; some subnuclear regions were spared of PABPC (Fig. 1B: viii, xii; Fig. 5B: iv, vii) This pattern was similar to the distribution of ZEBRA. The subnuclear regions spared of ZEBRA correspond to nucleoli, as identified by nucleolin as a marker [24] (Fig. 5A). To determine whether subnuclear regions spared of translocated PABPC also correspond to nucleoli, lytically-induced 2089 cells were co-stained with antibodies to nucleolin and PABPC. Subnuclear regions spared of translocated PABPC contained high concentrations of nucleolin (Fig. 5B). In lytically induced cells, nucleolin was partially dispersed and diffusely distributed throughout the nucleus (Fig. 5B: v, viii; blue arrows). This pattern of distribution of nucleolin suggests that during lytic EBV replication nucleolar structure is disrupted and nucleolar components are redistributed. When lytically induced 2089 cells were co-stained for BGLF5 and nucleolin, BGLF5 was present in concentrated foci, some of which over-lapped nucleoli (Fig. 5C: x-xv). In other cells, BGLF5 co-localized with large subnuclear globular regions enriched in dispersed nucleolin (Fig. 5C: xiii-xv). This pattern of distribution of BGLF5 differs from the distribution of ZEBRA and PABPC, both of which spare nucleoli (Fig. 5A, 5B).

**Figure 6:**
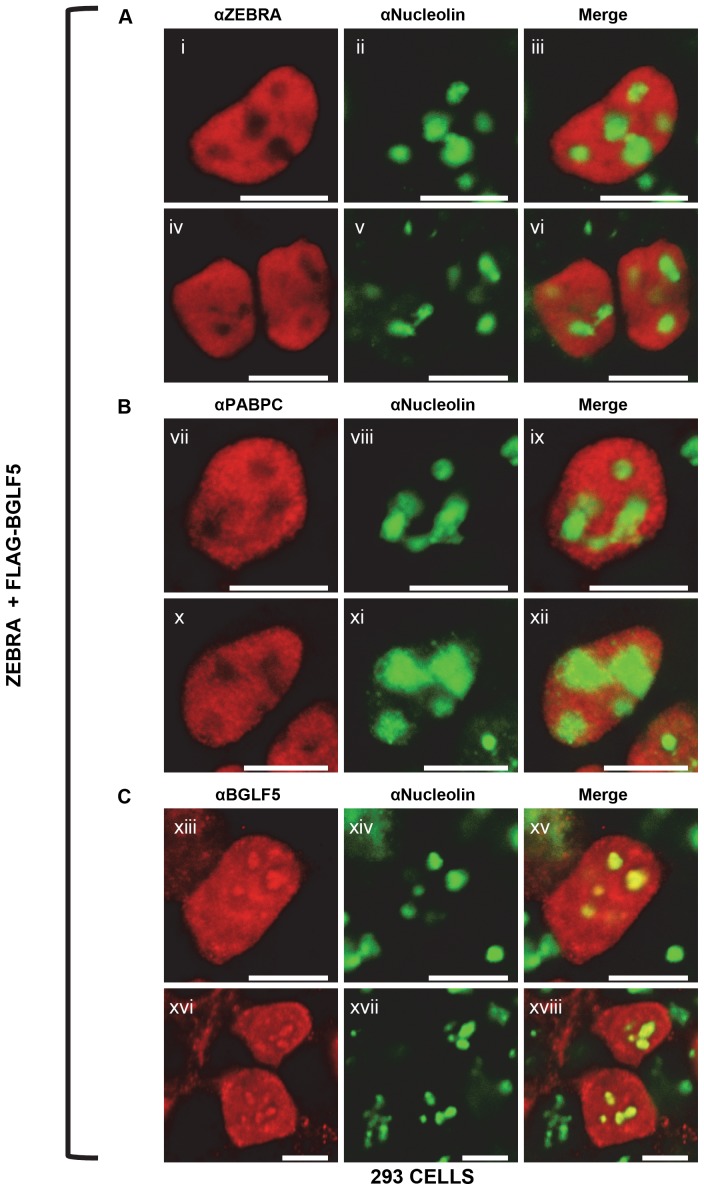
The intranuclear distributions of ZEBRA, PABPC and BGLF5 with respect to nucleolin are independent of other viral factors. 293 cells were co-transfected with ZEBRA and FLAG-BGLF5. Cells were fixed and stained with antibodies specific for ZEBRA, nucleolin, PABPC, or BGLF5, and fluorophore-conjugated secondary antibodies. Each of the following sets of panels depicts the same field of view: [i-iii], [iv-vi], [vii-ix], [x-xii], [xiii-xv], [xvi-xviii]. Reference bar in each panel equals 10 μM in length.

The distributions of ZEBRA, BGLF5, and translocated PABPC with respect to nucleoli seen during lytic induction are the same in cells lacking EBV. In 293 cells co-transfected with ZEBRA and BGLF5 and co-stained for ZEBRA and nucleolin, ZEBRA was distributed diffusely and exhibited its characteristic propensity to spare nucleoli (Fig. 6A). Cells co-stained for PABPC and nucleolin showed that translocated PABPC was also distributed diffusely and spared nucleoli (Fig. 6B). In 293 cells co-stained for BGLF5 and nucleolin, BGLF5 was enriched within nucleoli (Fig. 6C). These experiments indicate that in 293 cells with and without an EBV bacmid, ZEBRA and PABPC spare nucleoli whereas BGLF5 concentrates in nucleoli.

### BGLF5, BMLF1, and SC35, but not PABPC, are concentrated in replication compartments

PABPC was excluded from certain nuclear subregions present during lytic infection of EBV. These subregions were enriched in BGLF5 and nucleolin and contained viral replication compartments (Fig. 5C), indicating that they are critical sites of lytic viral activity. To examine further the composition of these PAPBC-deficient compartments and to investigate BGLF5's role in PABPC re-localization and host shutoff, we compared the localization of BGLF5 with respect to the viral proteins EA-D and Rta and the cellular protein SC35. SC35 is an RNA splicing factor whose intranuclear distribution in “nuclear speckles” has been well documented [26].

**Figure 7:**
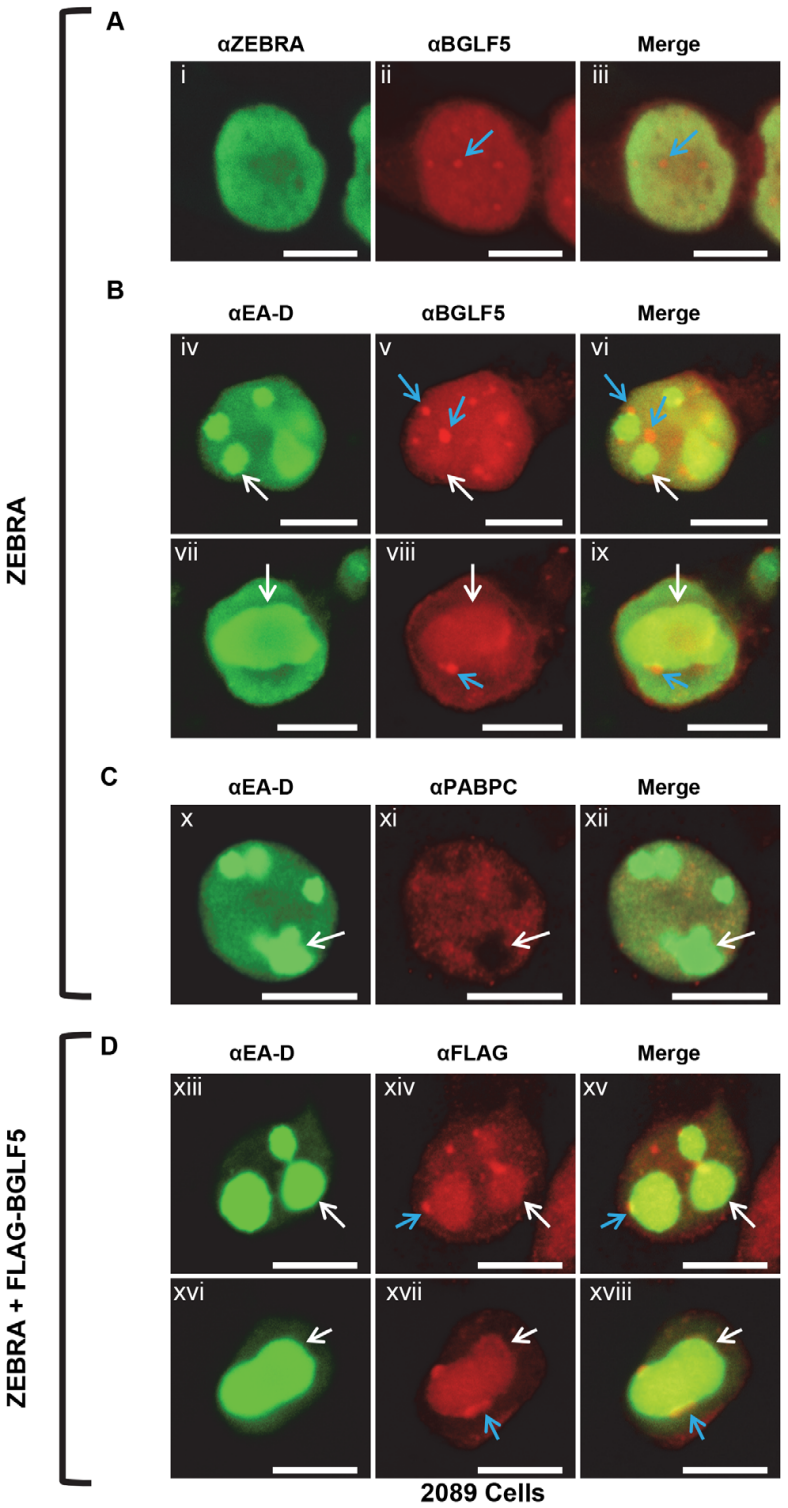
During EBV lytic replication, BGLF5 is recruited to viral replication compartments and to nodules at the periphery of viral replication compartments. 2089 cells were transfected with: (A, B, C) ZEBRA or (D) ZEBRA and FLAG-BGLF5. Cells were fixed and stained with antibodies specific for ZEBRA, BGLF5, EA-D, PABPC, and FLAG, and fluorophore-conjugated secondary antibodies. Each of the following sets of panels depicts the same field of view: [i-iii], [iv-vi], [vii-ix], [x-xii], [xiii-xv], [xvi-xviii]. Blue arrows indicate nodular foci of BGLF5. White arrows indicate globular viral replication compartments. Reference bar in each panel equals 10 μM in length.

In 2089 cells that were lytically induced by transfection of ZEBRA (Fig. 7A, 7B) or co-transfected with ZEBRA and FLAG-tagged BGLF5 (Fig. 7D), BGLF5 localized diffusely in the nucleus but was also present at lower levels in the cytoplasm (Fig. 7: ii, v, viii, xiv, xvii). In the nucleus, BGLF5 was concentrated within several (1-10) small nodule-like foci (Fig. 7: blue arrows). Co-staining with EA-D showed that some BGLF5 concentrated within globular viral replication compartments (Fig. 7B, 7D; white arrows) and some in nodules situated on the outer surface of viral replication compartments (Figs. 7B, 7D; blue arrows). This distribution of BGLF5 was markedly different from the sparing of viral replication compartments by translocated PABPC (Fig. 1B: xv-xvii, blue arrows; Fig. 7C: x-xii, white arrows).

**Figure 8:**
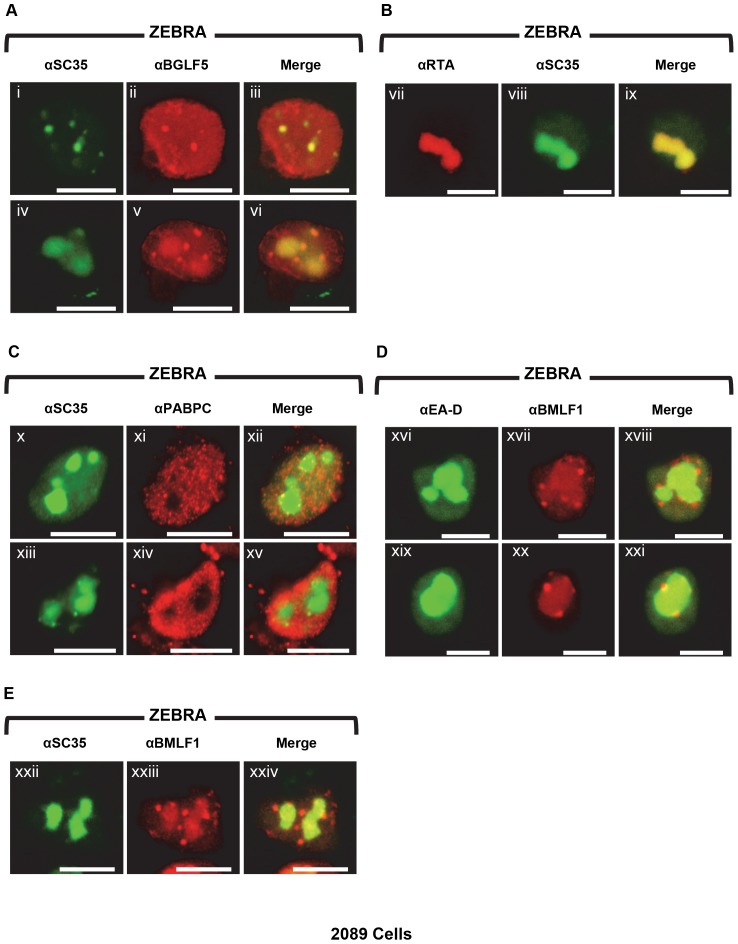
Translocated PABPC spares replication compartments but BGLF5, Rta, and SC35 co-localize in viral replication compartments. 2089 cells were transfected with ZEBRA. Cells were fixed and stained with antibodies specific for: BGLF5, SC35, Rta, BMLF1, and PABPC, and fluorophore-conjugated secondary antibodies. Each of the following sets of panels depicts the same field of view: [i-iii], [iv-vi], [vii-ix], [x-xii], [xiii-xv], [xvi-xviii], [xix-xxi], [xxii-xxiv]. Reference bar in each panel equals 10 μM in length.

During EBV lytic infection, SC35 co-localized with BGLF5 in punctate foci (Fig. 8A: i-iii) and within viral replication compartments (Fig. 8A: iv-vi). Co-localization of SC35 with viral replication compartments was verified by co-staining with Rta (Fig. 8B). Rta concentrates in replication compartments [24], [25]. Co-staining with PABPC showed that SC35 consistently localized to subnuclear regions characteristic of replication compartments that were spared of translocated PABPC (Fig. 8C).

EBV BMLF1 (also called EB2 or SM) exports viral mRNAs from the nucleus to the cytoplasm [27], [28]. Co-staining of EA-D and BMLF1 showed enrichment of BMLF1 within globular viral replication compartments. Concentrated nodules of BMLF1 were located on the surface of replication compartments (Fig. 8D). BMLF1 co-localized with SC35 (Fig. 8E). We conclude that PABPC is excluded from nuclear regions that contain viral replication compartments and adjacent nodules.

### Nuclear translocation of PABPC by ZEBRA is mechanistically distinct from regulation of intranuclear distribution of PABPC by ZEBRA

**Figure 9:**
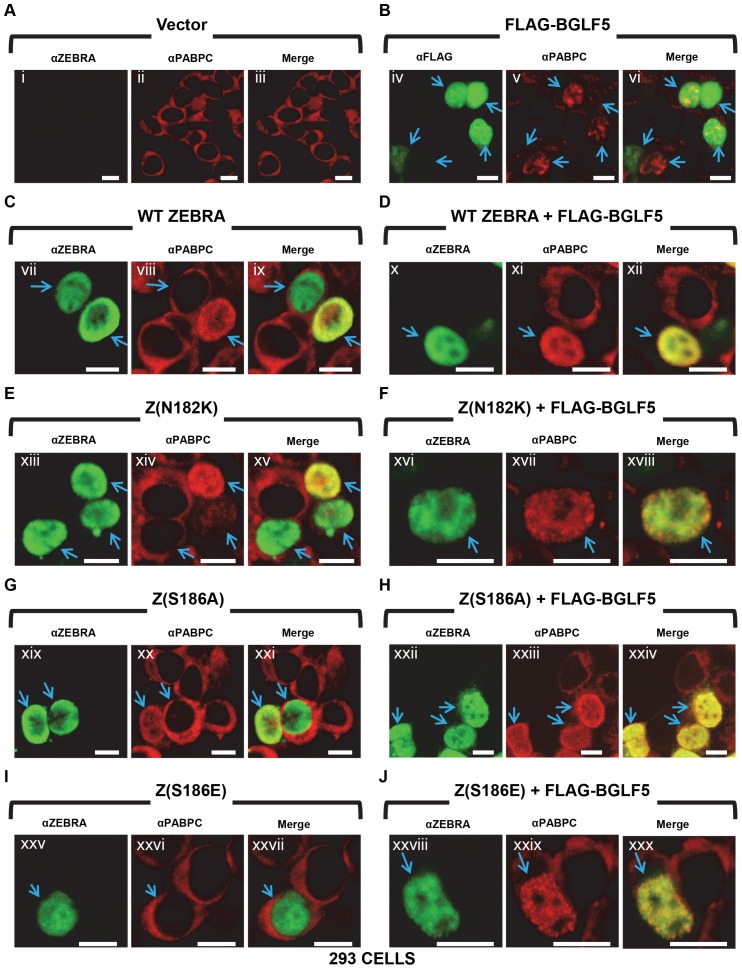
ZEBRA-induced translocation of PABPC and regulation of the intranuclear distribution of PABPC by ZEBRA are mechanistically distinct. 293 cells were transfected with empty vector or expression vectors for wild-type and mutant ZEBRA proteins without (panels A, C, E, G, I) or with FLAG-BGLF5 (panels B, D, F, H, J). Cells were fixed and stained with antibodies specific for ZEBRA and PABPC, and fluorophore-conjugated secondary antibodies. Each of the following sets of panels depicts the same field of view: [i-iii], [iv-vi], [vii-ix], [x-xii], [xiii-xv], [xvi-xviii], [xix-xxi], [xxii-xxiv], [xxv-xxvii], [xxviii-xxx]. Reference bar in each panel equals 10 μM in length.

To investigate mechanisms by which activities of ZEBRA regulate translocation and intranuclear distribution of PABPC, we used three point mutants of ZEBRA, Z(N182K), Z(S186A), and Z(S186E), each defective for transcriptional activation of downstream lytic viral genes [29], [30]. Z(N182K) and Z(S186E) are defective for binding the high affinity ZIIIB ZEBRA response element (ZRE) [30]. Although Z(S186A) efficiently binds to three ZREs, ZRE-R1, ZIIIA, and ZIIIB, as well as the AP-1 octamer site [31], this mutant is deficient in binding to methylated ZRE-R3 in the promoter of the EBV gene encoding Rta [32]. Transfection of Z(N182K) and Z(S186A) into 293 cells caused nuclear translocation of PABPC (Fig. 9E, 9G) in most cells expressing the mutant ZEBRA. Cell counts (Table 2) showed no significant decrease in PABPC translocation by Z(N182K) (58.6% of 133 cells) or by Z(S186A) (65.6% of 131 cells) compared to WT ZEBRA (60.9% of 174 cells). In contrast, ZEBRA mutant Z(S186E) (Fig. 9I; Table 2) showed a marked defect in translocation of PABPC (3.4% of 116 cells containing mutant ZEBRA) compared to WT ZEBRA.

**Table 2:** ZEBRA-mediated translocation of PABPC and regulation of the intranuclear distribution of translocated PABPC by ZEBRA are mechanistically distinct.

Transfection	Regulation of PABPC Nuclear Distribution	Cytoplasm to Nucleus Translocation of PABPC
Vector	−	−(***0%***)
ZEBRA	+	+(106/174: ***60.9%***)
Z(N182K)	+	+(78/133: ***58.6%***)
Z(S186A)	+	+(86/131: ***65.6%***)
Z(S186E)	+	−(4/116: ***3.4%***)
**293 CELLS**		

As described for Fig. 9, 293 cells transfected with empty vector or expression vectors for wild-type (WT) and mutant ZEBRA in the presence or absence of transfected BGLF5 were fixed and stained with antibodies specific for ZEBRA and PABPC. Cells expressing WT or mutant ZEBRA proteins were scored for ZEBRA-induced alterations to the intranuclear distribution of PABPC, and for ZEBRA-induced translocation of PABPC from the cytoplasm to the nucleus.

To assess the ability of the ZEBRA mutants to distribute PABPC re-localized within the nucleus, each was co-transfected with FLAG-BGLF5. Z(S186A) and Z(S186E) distribute evenly and diffusely in the nucleus similarly to WT ZEBRA; Z(N182K) is diffusely yet unevenly distributed. Some clumping of the Z(N182K) mutant was seen (Fig. 9F) [24], [33]. In contrast to the clumped distribution of intranuclear PABPC in cells transfected with BGLF5 alone (Fig. 9B), cells co-transfected with BGLF5 and each ZEBRA mutant showed a distribution pattern of PABPC that was identical to the respective ZEBRA mutant (Figs. 9F, 9H, 9J, S5). The mutant Z(N182K) conferred a diffuse yet uneven distribution pattern upon PABPC; this distribution was distinct from the uniformly diffuse distribution seen with WT ZEBRA, and was clearly different from the high degree of clumping seen with BGLF5. A ZEBRA mutant, Z(R183E), which induces nuclear aggregates and is concentrated in nuclear aggresomes [33], colocalized with PABPC within the nuclear aggregates and aggresomes when transfected in the absence of BGLF5 (Fig. S5A). When Z(R183E) was co-transfected with BGLF5, PABPC adopted a distribution similar to ZEBRA in aggresomes and small aggregates (Fig. S5B). These observations suggest that ZEBRA directs the distribution pattern of PABPC. The inability of Z(S186E) to translocate PABPC and its ability to efficiently direct the intranuclear distribution of PABPC indicates that these two functions of ZEBRA are distinct.

### Both ZEBRA and BGLF5 contribute to viral host shutoff

**Figure 10:**
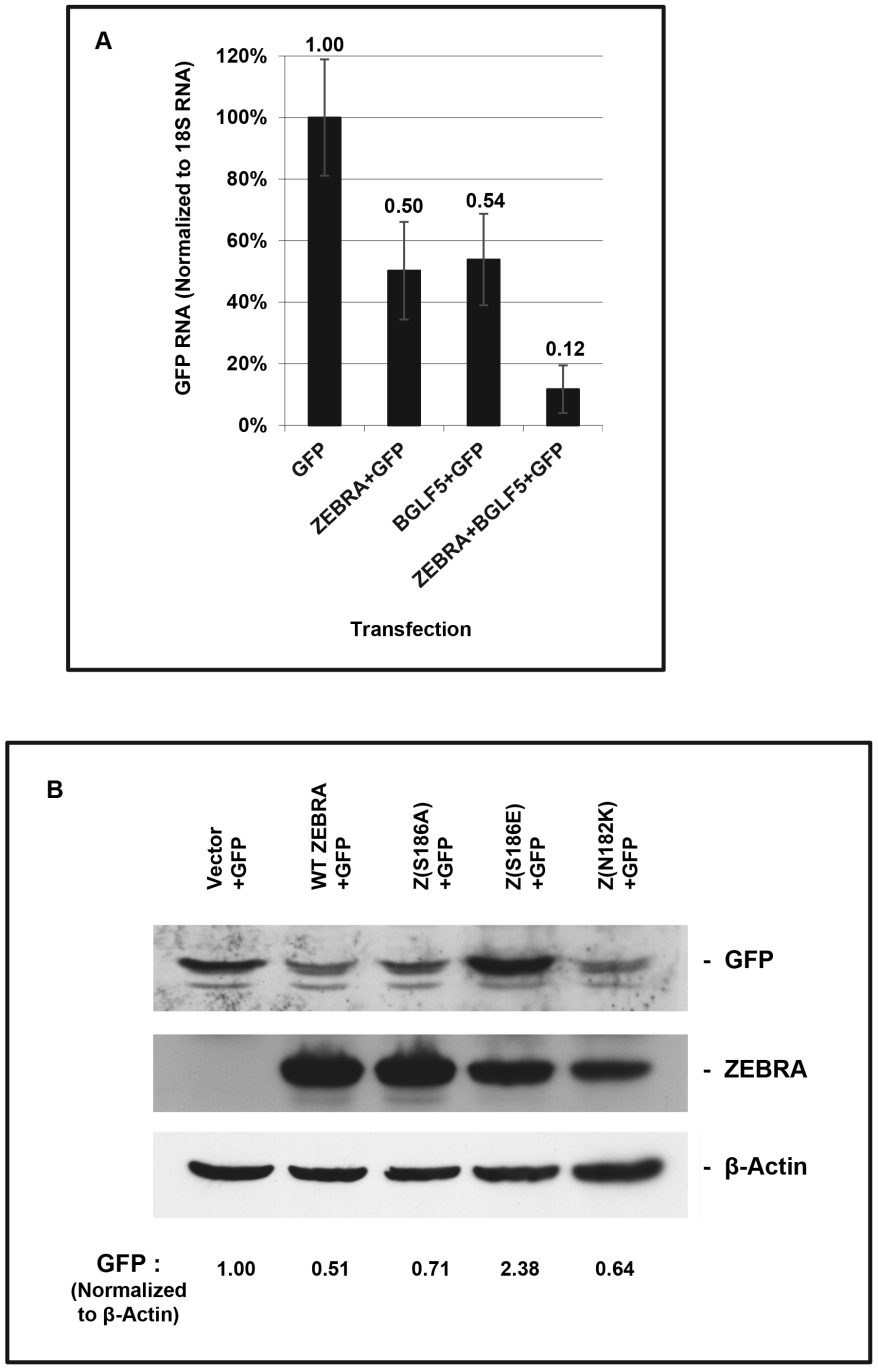
ZEBRA and BGLF5 decrease levels of GFP mRNA and protein; one point mutant of ZEBRA does not inhibit GFP expression. (A) 293 cells were transfected with pHD1013, or vectors expressing GFP, ZEBRA, or FLAG-BGLF5. RNA extracts were prepared 45 h after transfection. Real-time RT-PCR analysis was performed using primers specific for GFP and 18S rRNA. Real time RT-PCR values for GFP were normalized to 18S rRNA values. Error bars were derived from variation in values obtained from technical replicates performed in triplicate. (B) 293 cells were co-transfected with GFP and vector, ZEBRA, Z(S186A), Z(S186E), or Z(N182K). Cell extracts were prepared 45 h after transfection and analyzed by SDS-page. Immunoblots were probed with antibody specific for GFP, ZEBRA, and β-actin. The levels of GFP were quantified by densitometry and normalized to levels of β-actin.

Inhibition of exogenously expressed GFP is a commonly used assay for EBV- and KSHV-induced host shutoff [15], [16], [18]. We measured the effects of ZEBRA and BGLF5 on levels of humanized renilla GFP mRNA by real-time RT-PCR (Fig. 10A); technical replicates were done in triplicate. Co-transfection of ZEBRA with GFP into 293 cells reduced the level of GFP mRNA by 50%. Co-transfection of BGLF5 and GFP reduced the level of GFP mRNA by 46%. Co-transfection of both ZEBRA and BGLF5 with GFP decreased GFP mRNA by 88%. To measure the effect of ZEBRA on GFP protein, and to correlate the ZEBRA-mediated translocation of PABPC with shutoff, WT ZEBRA, Z(N182K), Z(S186A), and Z(S186E) were co-transfected with GFP. WT ZEBRA reduced expression of GFP by 49% compared to the vector control (Fig. 10B). Z(N182K) and Z(S186A) reduced expression of GFP by 36%, and 29%, respectively, compared to the vector control (Fig. 10B). In contrast, Z(S186E), which was defective for PABPC translocation (Fig. 9I; Table 2), did not reduce expression of GFP (Fig. 10B). Thus WT ZEBRA inhibited expression of GFP mRNA and protein, and a mutation in ZEBRA that prevented PABPC translocation also suppressed ZEBRA's ability to reduce expression of GFP.

**Figure 11:**
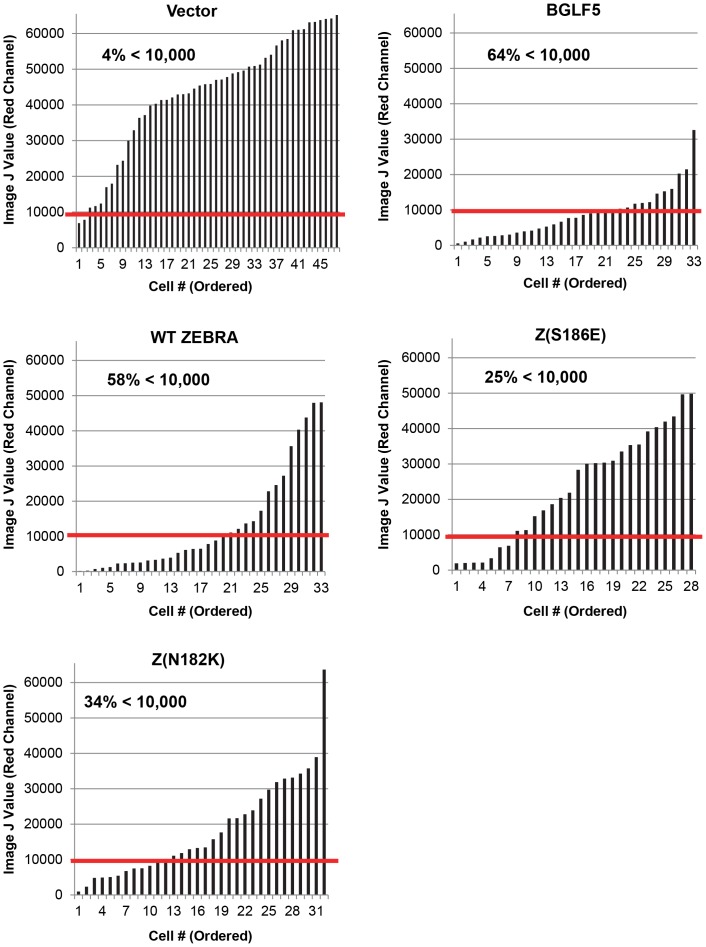
BGLF5 and ZEBRA function as viral host shutoff factors that inhibit endogenous expression of host genes on a global scale; point mutations impair ZEBRA's host shutoff activity. 293 cells were transfected with pHD1013, or vectors expressing BGLF5, ZEBRA, Z(N182K), or Z(S186E). Cells were incubated in methionine-free, cysteine-free media containing HPG, then fixed. Using click-chemistry based reagents, incorporated HPG was covalently bound to Alexa Fluor 555. Cells were stained with antibodies specific for ZEBRA and lamin B, and fluorophore-conjugated secondary antibodies. Images were acquired by confocal microscopy. For each population of transfected cells, levels of newly synthesized proteins in individual cells was quantitatively measured using ImageJ software (NIH) analysis of the intensity of red channel emissions. ImageJ values were plotted in increasing order and the percentage of cells below 10,000 (red line) was calculated.

ZEBRA-mediated translocation of PABPC and inhibition of GFP expression suggest that ZEBRA plays a role in vhs. To investigate further ZEBRA's ability to function as a viral host shutoff factor, we assayed for ZEBRA's ability to reduce endogenous expression of host proteins on a global scale. Using commercially available reagents that utilize click chemistry to covalently bind fluorophores to a methionine analog incorporated into newly synthesized proteins, new protein synthesis was imaged by confocal microscopy then quantitatively measured at the single cell level by ImageJ analysis. Coupled with immunofluorescence analysis, this technique allowed measurement of differences in new protein synthesis in individual cells expressing a given protein of interest. 293 cells transfected with empty vector, or expression vectors for BGLF5, WT ZEBRA, Z(N182K), or Z(S186E) were analyzed for new protein synthesis (Fig. S6; Fig. 11; Table 3).

**Table 3:** BGLF5 and ZEBRA Induce Viral Host Shutoff; Point Mutations Impair ZEBRA's Host Shutoff Activity.

Transfection	# Cells	ImageJ Measurements				
		Range	AVG (MEAN)	AVG(MEAN; %)	% Cells<10,000	p-Value(Vector Comparison)
Vector	48	6888–65,180	43214	100%	4%	*
BGLF5	33	554–32,584	8788	20%	64%	1.46549E-13
WT ZEBRA	33	189–48,090	13285	31%	58%	9.78155E-11
Z(S186E)	28	1923–49815	23545	54%	25%	1.24268E-06
Z(N182K)	32	954–63,680	18325	42%	34%	3.16786E-09

Data shown in table represents results depicted in Fig. 11. Mean averages were calculated as the quotient of ImageJ measurements of red channel (HPG; Alexa Fluor 555) emissions of individual cells divided by the number of cells for each transfection condition. Statistical analysis was performed using the Mann-Whitney U test to compare differences in ImageJ measurements between the transfected protein and the vector control.

Cells transfected with the vector control showed relatively high levels of new protein synthesis, with recently synthesized proteins localizing primarily to the cytoplasm but also localizing significantly to the nucleus (Fig. S6: i-iv). Cells transfected with BGLF5 showed markedly decreased levels of new protein synthesis (Fig. S6: v-viii; blue arrows). Cells expressing ZEBRA also showed a significant decrease in new protein synthesis (Fig. S6: ix-xvi; blue arrows). Cells containing relatively low levels of WT ZEBRA (Fig. S6, xiii-xvi, yellow arrows) were capable of reducing new protein synthesis as efficiently as individual cells containing high levels (Fig. S6, xiii-xvi, purple arrows). This result indicates that a correlation does not exist between expressed levels of ZEBRA and the degree of host shutoff. Both BGLF5 and ZEBRA cause significant global shutdown of host protein synthesis. The Z(S186E) and Z(N182K) mutants also showed significant decreases in new protein synthesis (Fig. S6: xvii-xxiv), although qualitatively reductions in protein synthesis were less than seen with BGLF5 and WT ZEBRA.

Three parameters derived from ImageJ measurements of approximately 30 randomly selected cells from each group of transfected cells were used to quantitate shutoff of host protein synthesis. These parameters included the mean value of HPG incorporation intensity per individual cell (Table 3), the distribution of values (Fig. 11), and the fraction of cells below a cut-off value (Fig. 11; Table 3). All three parameters showed that BGLF5 caused the greatest inhibition of new protein synthesis, followed by ZEBRA. The mutants Z(N182K) and Z(S186E) each caused a statistically significant decrease in new protein synthesis compared to the vector (Table 3). Z(S186E), which was most impaired in host shutoff, was statistically significantly different compared to WT ZEBRA (p value<0.0057) (Table 4).

**Table 4:** Defect in new protein synthesis by the Z(S186E) mutant is significant.

Statistical Comparison	p-Value
WT ZEBRA vs. Z(S186E)	0.0056581566
WT ZEBRA vs. Z(N182K)	0.0203305575

Data shown in table represents statistical analysis of results depicted in Fig. 11. Mann-Whitney U test was used to compare differences in mean averages of ImageJ measurements between wild-type and mutant ZEBRA.

## Discussion

### Novel insights into regulation of PABPC localization and vhs during lytic EBV infection

This report describes novel functions of the EBV lytic cycle activator protein, ZEBRA, in translocation and regulation of nuclear distribution of PABPC. These function are consistent with a role of ZEBRA in mediating widespread inhibition of cellular protein synthesis. In EBV-infected cells, translocation of PABPC begins during the early stage of lytic infection in cells lacking replication compartments (Table 1). Translocation of PABPC is mediated by BGLF5 and ZEBRA, two early viral proteins that are each sufficient to mediate translocation of PABPC without the involvement of other viral proteins (Figs. 3, 4). BGLF5 and ZEBRA play distinct roles in the nuclear distribution of PABPC. In the absence of ZEBRA, BGLF5 distributes translocated PABPC in a clumpy pattern within the nucleus rather than in the diffuse pattern seen during lytic induction (Fig. 3). ZEBRA directs the intranuclear distribution of PABPC into a diffuse pattern. Although ZEBRA by itself induces some translocation of PABPC in the absence of BGLF5, translocation of PABPC was maximal when ZEBRA and BGLF5 were expressed together (Fig. 4). Intranuclear PABPC co-localizes with ZEBRA, not with BGLF5. PABPC is excluded from regions of the nucleus corresponding to nucleoli, globular viral replication compartments and nodular foci that accumulate BGLF5, BMLF1, and SC35 (Figs. 5–8). ZEBRA and BGLF5 each individually inhibit expression of a reporter of host cell shutoff, GFP, at both the mRNA and protein levels. When ZEBRA and BGLF5 are expressed together, inhibition of GFP expression is maximal (Fig. 10). Both ZEBRA and BGLF5 globally inhibited cellular protein synthesis when assessed by click chemistry (Fig. S6; Fig. 11; Table 3). A ZEBRA mutant, Z(S186E), that is deficient in translocation of PABPC did not by itself inhibit expression of GFP in the shutoff reporter assay (Fig. 9; Fig. 10; Table 2). This mutant was also significantly impaired in its ability to inhibit protein synthesis (Table 4). ZEBRA is well known as a transcriptional activator of early EBV genes and as an essential replication protein that binds to the lytic origin of replication [34], [35]. Regulation of cellular protein localization within the nucleus and a direct role in viral host shutoff are novel functions for ZEBRA.

### Mechanisms of vhs in alpha- and gamma- herpesviruses

The *vhs* protein, the primary inducer of host shutoff by HSV-1, is an RNA endonuclease that directly and efficiently degrades all cellular mRNAs during the immediate early and early stages of lytic viral infection [11], [36]. *Vhs* also induces translocation of PABPC to the nucleus, whereas a host-shutoff-defective mutant of *vhs* does not translocate PABPC [12]. Host shutoff and translocation of PABPC to the nucleus are also regulated by HSV-1 ICP27, a multifunctional immediate-early protein with roles in transcription, mRNA splicing, mRNA nuclear egress, and translation [13]. ICP27 binds RNA, interacts with several splicing factors, causes a redistribution of splicing factors, and inhibits splicing of host RNAs [37]-[43], thereby reducing levels of cytoplasmic spliced mRNAs.

Gammaherpesviruses mediate global mRNA decay and translocation of PABPC using conserved viral proteins, KSHV SOX, EBV BGLF5, and MHV68 muSOX, which are alkaline nucleases [15], [16], [18], [20], [44]. Rather than directly catalyzing global mRNA degradation in the manner of HSV-1 *vhs*, KSHV SOX introduces site-specific cleavage within mRNAs. An efficient cellular RNA exonuclease, Xrn1 recognizes the cleavage site [17]. Xrn1-mediated mRNA decay liberates PABPC from mRNA in the cytoplasm, thereby exposing importin α binding sites within the RNA recognition motifs of PABPC [17]. Binding of PABPC to importin α leads to translocation of PAPBC into the nucleus. BGLF5 and muSOX may induce translocation of PABPC through a similar mechanism involving Xrn-1 [19].

Translocation of PABPC from the cytoplasm into the nucleus and establishment of the intranuclear distribution of PABPC during lytic EBV infection are distinct functions that are mediated by two viral proteins. This mechanism of PABPC relocalization by two EBV proteins differs from the mechanisms of host shutoff and PABPC relocalization that are mediated by a single protein, SOX or muSOX, during infections of KSHV or MHV68. For EBV, translocation of PABPC from the cytoplasm to the nucleus is mediated by BGLF5 and by ZEBRA. A diffuse intranuclear distribution of PABPC characteristic of lytic infection is directed by ZEBRA alone. In the absence of ZEBRA, translocated PABPC mediated by BGLF5 appears in clumps that are unlike the distribution of PABPC during lytic infection. BGLF5 neither co-localized with PABPC, nor did BGLF5 distribute PABPC diffusely (Figs. 2C, 3C, 3D). ZEBRA, however, co-localized with PABPC and conferred the diffuse distribution seen during lytic induction, when transfected alone or with BGLF5. ZEBRA co-localized exactly with PABPC throughout the nucleus with the sole exception of globular viral replication compartments. ZEBRA localized to replication compartments, whereas PABPC was excluded, probably because ZEBRA plays a direct role in lytic viral DNA replication [35].

### Correlation between vhs and PABPC relocalization during EBV lytic infection

We found that like BGLF5 ZEBRA also down-regulates expression of GFP mRNA and protein, and enhances the shutoff effect of BGLF5 on GFP (Fig. 10). Moreover, ZEBRA and BGLF5 also block endogenous protein synthesis (Fig. S6; Fig. 11; Table 3). These findings support a role for ZEBRA in EBV host shutoff. ZEBRA's capacity to translocate PABPC is an essential component of host shutoff. The Z(S186E) mutant that is deficient in PABPC translocation does not inhibit GFP expression and is impaired in shutoff of protein synthesis. The model of shutoff from studies of KSHV proposes that hyperadenylated mRNAs sequestered within the nucleus directly associate with translocated PABPC [12]. ZEBRA's role in ensuring a diffuse distribution of PABPC that encompasses the entire nuclear volume may also be important for maximal sequestration of hyperadenylated mRNAs.

### Subnuclear regions spared of translocated PABPC may selectively rescue viral functions from shutoff

A key objective of host shutoff is to achieve efficient viral gene expression by reallocation of cellular resources. Therefore, vhs mechanisms targeting PABPC as a means of suppressing host gene expression must allow processes that selectively permit viral genes to continue to function efficiently. Viral targeting of PABPC plays a role in selective expression in other viruses. For instance, rotavirus transcriptase synthesizes viral mRNAs that are capped but not polyadenylated. These mRNAs possess a 3′- terminal sequence that binds NSP3. Eviction of PABPC from mRNAs by NSP3 and nuclear relocalization of PABPC shuts down host protein synthesis. However, NSP3 bound to 3′-termini of viral mRNAs functionally replaces PABPC by binding eIF4G and thereby selectively promotes translation of viral mRNAs [45], [46]. In another example, vaccinia virus (VV) mRNAs are capped and polyadenylated; however, translation of host mRNAs is strongly suppressed during VV infection whereas translation of viral mRNAs are not. Selective translation of VV mRNAs is conferred by dramatic redistribution of translation initiation factors eIF4E, eIF4G, and PABPC to discrete viral replication factories in the cytoplasm where viral transcription and translation occur [47].

EBV mRNAs are capped and polyadenylated and would be subject to hyperadenylation and retention in the nucleus upon binding of translocated PABPC. However, we consistently observed distinct nuclear sub-regions devoid of PABPC interspersed within diffusely distributed translocated PABPC. Presumably, sequestration of mRNAs and a block to their export from the nucleus would not occur at these sites lacking PABPC. We found that regions spared of PABPC include viral replication compartments containing the cellular RNA splicing factor, SC35, nucleolin, and three viral proteins, Rta, BGLF5, and the viral RNA export factor, BMLF1 (Figs. 1, 5, 8). These findings support the idea that, in addition to being sites of viral DNA replication, these compartments spared of PABPC may also be sites of viral late gene transcription [48], RNA processing, and locales for selective licensing for export of viral mRNAs. Similar sites from which PABPC is excluded are seen in nuclei of cells infected with KSHV, HSV-1, or rotavirus [9], [12], [13]. Thus, the distribution of PABPC within the nucleus, as controlled by ZEBRA, may constitute a means of selectively enabling viral mRNAs to evade the shutoff mechanism.

## Materials and Methods

### Cell lines

HH514-16 is a subclone of the P3J-HR1K Burkitt lymphoma cell line [49], [50]. 293 is a human embryonic kidney cell line immortalized by the early region of adenovirus [51]. 2089 is a 293 cell line stably transfected with a bacmid containing the B95-8 EBV genome and a hygromycin B-resistance gene [21]. BZKO is a 293 cell line containing an EBV-bacmid in which the BZLF1 gene has been inactivated by insertion of a kanamycin resistance cassette [22]. BGLF5-KO is a 293 cell line containing an EBV-bacmid in which part of the BGLF5 gene was replaced with a kanamycin resistance cassette [23]. 293 cells were maintained in RPMI 1640 complete media, supplemented with 10% fetal bovine serum, 50 units/mL penicillin-streptomycin, and 1 ug/mL amphotericin B. 2089 cells, BZKO cells, and BGLF5-KO cells were maintained in RPMI 1640 complete media containing 100 ug/mL hygromycin B (Calbiochem).

### Antibodies

In immunofluorescence and immunoblotting experiments, ZEBRA was detected with a rabbit polyclonal antibody (S1605) or BZ1 mouse monoclonal antibody [52]. S1605 was prepared from rabbits immunized with full length ZEBRA protein which was expressed in *E. coli* from a pET22b vector containing the BZLF1 cDNA and purified over a nickel-agarose column. Rta was detected using rabbit polyclonal antisera described previously [30]. EA-D was detected using the mouse monoclonal antibody R3.1 [53]. BGLF5 was detected using a rabbit polyclonal antibody prepared from rabbits immunized with nearly full-length (amino acids 2 – 469) BGLF5 protein expressed in *E. coli* from a pET22 vector containing the corresponding encoding sequence of the BGLF5 gene. β-actin was detected using a mouse monoclonal antibody purchased from Sigma (A5316). SC35, nucleolin, and tubulin proteins were detected using mouse monoclonal antibodies purchased from Abcam (ab11826; ab13541; ab7291). hr-GFP was detected using a rabbit polyclonal antibody purchased from Stratgene (#240142-51). Lamin B was detected using a goat polyclonal antibody purchased from Santa Cruz Biotech. (sc-6216). FLAG-tag was detected using a mouse monoclonal antibody purchased from Sigma (# F1804). Secondary antibodies used in immunofluorescence experiments were purchased from Jackson ImmunoResearch Labs: FITC-sheep anti-mouse IgG (#515-095-062), Texas Red-donkey anti-rabbit IgG (#711-075-152), FITC-donkey anti-goat IgG (#705-095-147), Rhodamine Red X-donkey anti-rabbit IgG (#711-295-152), DyLight 549-donkey anti-rabbit IgG (#711-505-152), Alexa Fluor 488-donkey anti-mouse (#715-545-150), Cy3-donkey anti-rabbit (#711-165-152), Alexa Fluor 647 donkey anti-goat (#805-605-180).

### Indirect immunofluorescence

2089, BGLF5-KO, and 293 cells grown on glass coverslips were transfected with plasmid DNA using DMRIE-C reagent (Invitrogen). After 8 hours the transfection reagent was replaced with growth media. Thirty-eight to forty-three hours after transfection, a time previously determined to be adequate for detection of lytic viral DNA replication, cells were fixed in chilled methanol for 30 min. at −20°C, washed with PBS, and incubated in blocking solution (10% human serum in PBS) for 1 hour at room temperature. Cells were stained with primary antibody diluted in blocking solution for 1 hour at room temperature in humidified chambers. Cells were washed with PBS, then incubated with secondary antibody diluted 1∶200 in blocking solution for 1 hour at room temperature in opaque humidified chambers. Cells were washed with PBS, briefly rinsed in distilled H_2_O to remove salts, then mounted on glass slides using Vectashield mounting media (Vector Laboratories). A Zeiss LSM510 confocal laser scanning microscope was used to obtain digital images of fluorescence and transmitted light.

### Assay for New Protein Synthesis

293 cells grown on glass coverslips were transfected with plasmid DNA using DMRIE-C reagent (Invitrogen). At forty hours post-transfection, cells were assayed for new protein synthesis using the commercially available Click-iT (Invitrogen) assay system of new protein synthesis according to the manufacturer's instructions. Briefly, cells were incubated in methionine-free, cysteine free DMEM media (MFCF-DMEM; Gibco #21013-024) supplemented with L-glutamine for 30–45 min at 37^o^ celsius. Cells were then incubated for 4 hours in MFCF-DMEM containing the methionine analog L-homopropargylglycine (Invitrogen; Cat#: C10186). Cells were fixed in chilled methanol, washed with PBS, and incubated in Click-iT reaction cocktail (Invitrogen; Cat#: C10269) containing Alexa Fluor 555 Azide (Invitrogen; Cat#: A20012), which covalently bound the alkyne group of HPG to the azide group of the fluorophore. Cells were washed with PBS, and processed for indirect immunofluorescence staining as described above. Digital images of transfected cells were acquired by confocal microscopy with equivalent photomultiplier acquisition settings for the red channel. To ensure randomness in selection of transfected cells, images were taken by observation of the green (transfected protein) and blue (lamin B) emissions only. The observer was blinded to red (HPG) channel emissions. New protein synthesis of single cells was quantitatively measured using ImageJ software (NIH) analysis of the intensity of red channel emissions. The Mann-Whitney U test was used to calculate p-values in comparisons of differences in ImageJ measurements for each transfected protein with the vector control measurements.

### Immunoblot Analysis

After 48 h of incubation at 37°C, BZKO cells were removed from the plastic surface by forceful pipetting, pooled, centrifuged, and resuspended in PBS. The cell suspension was divided into five tubes and spun down. Each cell pellet was flash frozen. To assay viral proteins, one pellet, containing 2×10^6^ cells, was resuspended in 40 μl SDS sample buffer. Samples were sonicated for 30 s and heated to 100°C for 5 min. Forty microliters was loaded per lane of a 10% SDS-polyacrylamide gel. After electrophoresis, the proteins were transferred to a nitrocellulose membrane by electroblotting for 30 min at 15 V using a Bio-Rad Transblot semidry transfer cell. The blots were blocked with 5% nonfat dry milk for 1 h and incubated for 1 to 2 h with human, rabbit, or murine antibodies to EBV lytic cycle proteins diluted in 5% nonfat dry milk. The blots were washed twice in Tris saline (TS) (10 mM Tris, pH 7.5, 200 mM NaCl, 5% Tween 20), incubated for 1 to 2 h with secondary antibodies appropriate for the species diluted in 5% nonfat dry milk, and washed twice in TS. To detect immunoreactive bands, blots were incubated with 1 μCi ^125^I-protein A (Amersham) in nonfat dry milk for 1 h and washed twice. The blots were exposed overnight with intensifying screens to Kodak XAR-5 film at −70°C.

293 cells were trypsinized and harvested 43 hours after transfection. Cells were washed once with cold PBS, pelleted, and resuspended in SDS sample buffer. Samples were sonicated for 1 min. and heated to 100°C for 5 min. Samples were electrophoresed on a 10% SDS-polyacrylamide gel. After electrophoresis, proteins were transferred from the gel to a nitrocellulose membrane. Blots were blocked overnight at 4°C in blocking solution (5% nonfat dry milk in TBS-T: 20 mM Tris, pH 7.5, 137 mM NaCl, 0.1% Tween 20), then incubated for 1 h with primary antibodies in blocking solution. The blots were washed in TBS-T, incubated for 1 h with horseradish peroxidase-conjugated secondary antibodies appropriate for the species diluted in blocking solution, and washed again in TBS-T. Immunoreactive bands were detected using a ECL chemiluminescence kit (GE: RPN 2106) performed according to manufacturer's recommended protocol.

### Quantitative RT-PCR

RNA was purified from 293 cells 43 hours after transfection using Qiagen products. The level of EBV transcripts encoding lytic viral replication proteins was determined using the iScript SYBR green RT-PCR kit (Bio-Rad). The amount of RNA present in each sample was normalized to 18S ribosomal RNA. Assays on individual samples were performed in triplicate. Error bars were derived from variation in values obtained from technical replicates. The efficiency of each primer set was determined by quantitative PCR using 10-fold serial dilution of template DNA. The following DNA sequences were used as primers to detect hr-GFP: forward 5′-CAAGTTCTACAGCTGCCACA-3′ and reverse 5′-TCCACGTAGGTCTTCTCCAG-3′, and 18S ribosomal RNA: forward 5′-GTAACCCGTTGAACCCCATT-3′ and reverse 5′-CCATCCAATCGGTAGTAGCG-3′.

## Supporting Information

Figure S1 **Induction of the EBV lytic cycle in Burkitt lymphoma cells is accompanied by translocation of PABPC from the cytoplasm to the nucleus.** HH514-16 cells were induced into the lytic phase by treatment with sodium butyrate. Cells were fixed and then stained with DAPI and with antibodies specific for EA-D (ii, v) and PABPC (iii, vi), and fluorophore-conjugated secondary antibodies. Digital images were acquired by confocal microscopy. Panels [i-iii] and [iv-vi] depict the same field of view. Arrows in panels [v, vi] denote cells undergoing viral lytic induction.
(TIF)

Figure S2 **Levels of PABPC during induction of the lytic phase, and during expression of ZEBRA and BGLF5.** (A) BZKO cells were transfected with vector (pHD1013) or pCMV-gZ expressing wild type ZEBRA. Cell extracts were prepared 48 h after transfection. Immunoblots were probed with antibodies to ZEBRA, PABPC and tubulin. (B) 293 cells were transfected with vector, ZEBRA or FLAG-BGLF5. Cell extracts were prepared 43 h after transfection. Immunoblots were probed with antibodies to FLAG, PABPC and β-actin.
(TIF)

Figure S3 **Rta does not redistribute intranuclear PABPC.** 293 cells were transfected with Rta and FLAG-BGLF5. Cells were fixed and stained with antibodies specific for PABPC and Rta, and fluorescent secondary antibodies. Reference bar in each panel equals 10 μM in length.
(TIF)

Figure S4 **ZEBRA but not c-Jun relocalizes FLAG-PABPC.** 293 cells were co-transfected with: (A) FLAG-PABPC, (B) ZEBRA and FLAG-PABPC, (C) c-Jun and FLAG-PABPC. Cells were fixed and stained with antibodies specific for ZEBRA, FLAG, and c-Jun, and fluorophore-conjugated secondary antibodies. Each of the following sets of panels depicts the same field of view: [i-iii], [iv-vi], [vii-ix], [x-xii], [xiii-xv], [xvi-xviii]. Reference bar in each panel equals 10 μM in length.
(TIF)

Figure S5 **The DNA-binding deficient aggresome-inducing mutant of ZEBRA, Z(R183E), relocalizes PABPC.** 293 cells were (A) transfected with Z(R183E) or (B) co-transfected with Z(R183E) and FLAG-BGLF5. Cells were fixed and stained with antibodies specific for ZEBRA and PABPC, and fluorophore-conjugated secondary antibodies. Each of the following sets of panels depicts the same field of view: [i-iii], [iv-vi], [vii-ix]. Reference bar in each panel equals 10 μM in length.
(TIF)

Figure S6 **BGLF5 and ZEBRA inhibit endogenous nascent protein synthesis on a global scale; point mutations in the basic region impair ZEBRA's host shutoff activity.** 293 cells were transfected with pHD1013, or vectors expressing BGLF5, ZEBRA, Z(N182K), or Z(S186E). Cells were incubated in methionine-free, cysteine-free media containing HPG, then fixed. Using click-chemistry based reagents, incorporated HPG was covalently bound to Alexa Fluor 555. Cells were stained with antibodies specific for ZEBRA and lamin B, and fluorophore-conjugated secondary antibodies. (A) Each of the following sets of panels depicts the same field of view: [i-iv], [v-viii], [ix-xii], [xiii-xvi], [xvii-xx], [xxi-xxiv]. Blue arrows denote cells expressing transfected protein. In panels [xiii-xvi], purple arrows denote cells expressing relatively high levels of ZEBRA, yellow arrows denote cells expressing relatively low levels of ZEBRA. Reference bar in each panel equals 10 μM in length.
(TIF)
