# Supplementary figures and images for: Nuclear Translocation and Regulation of Intranuclear Distribution of Cytoplasmic Poly(A)-Binding Protein Are Distinct Processes Mediated by Two Epstein Barr Virus Proteins
Source: PLoS One. 2014 Apr 4;9(4):e92593. doi: 10.1371/journal.pone.0092593 (PMC3976295; doi:10.1371/journal.pone.0092593)

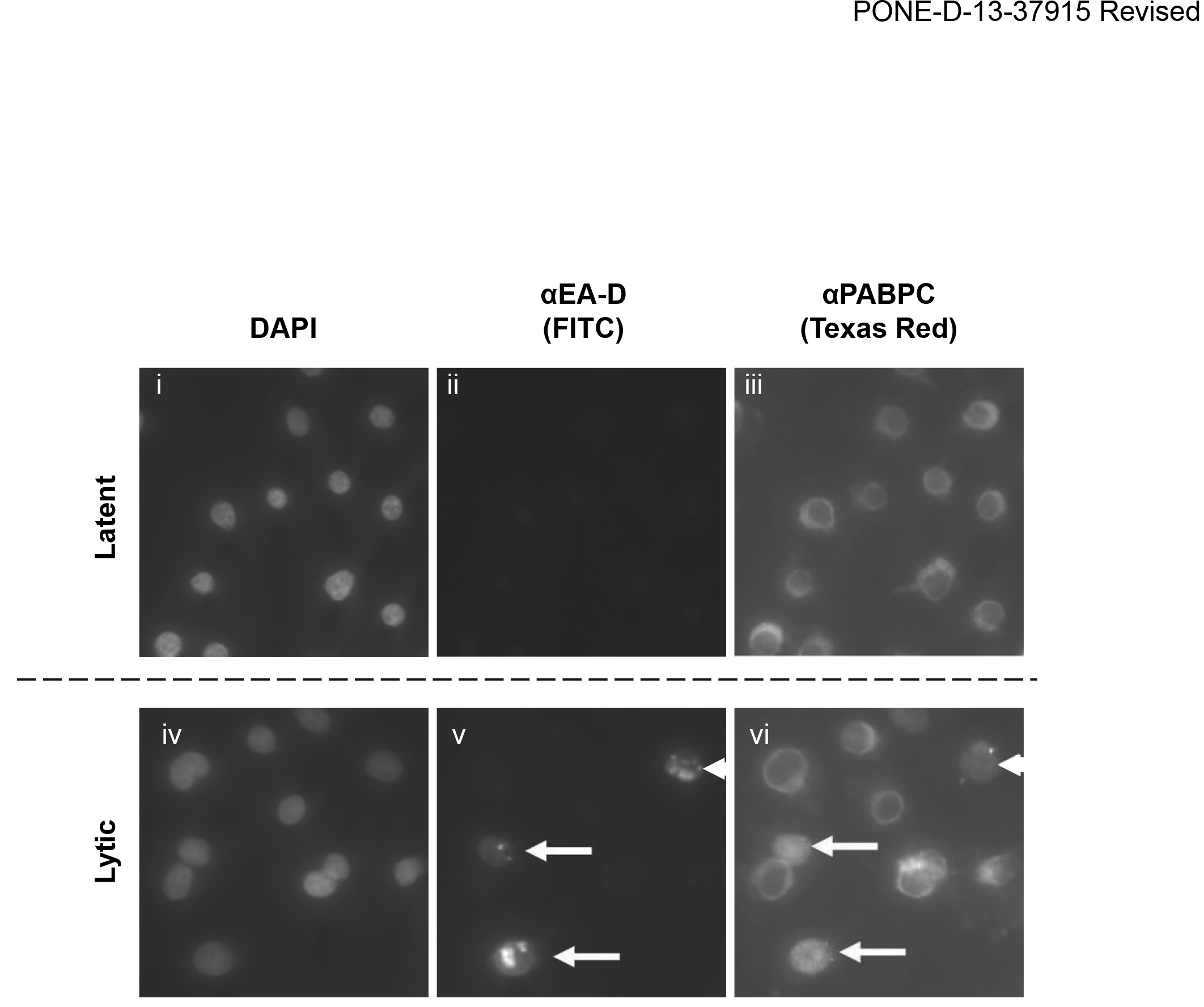

Supplement: Figure S1 — Induction of the EBV lytic cycle in Burkitt lymphoma cells is accompanied by translocation of PABPC from the cytoplasm to the nucleus. HH514-16 cells were induced into the lytic phase by treatment with sodium butyrate. Cells were fixed and then stained with DAPI and with antibodies specific for EA-D (ii, v) and PABPC (iii, vi), and fluorophore-conjugated secondary antibodies. Digital images were acquired by confocal microscopy. Panels [i-iii] and [iv-vi] depict the same field of view. Arrows in panels [v, vi] denote cells undergoing viral lytic induction. (TIF) [file pone.0092593.s001.tif]

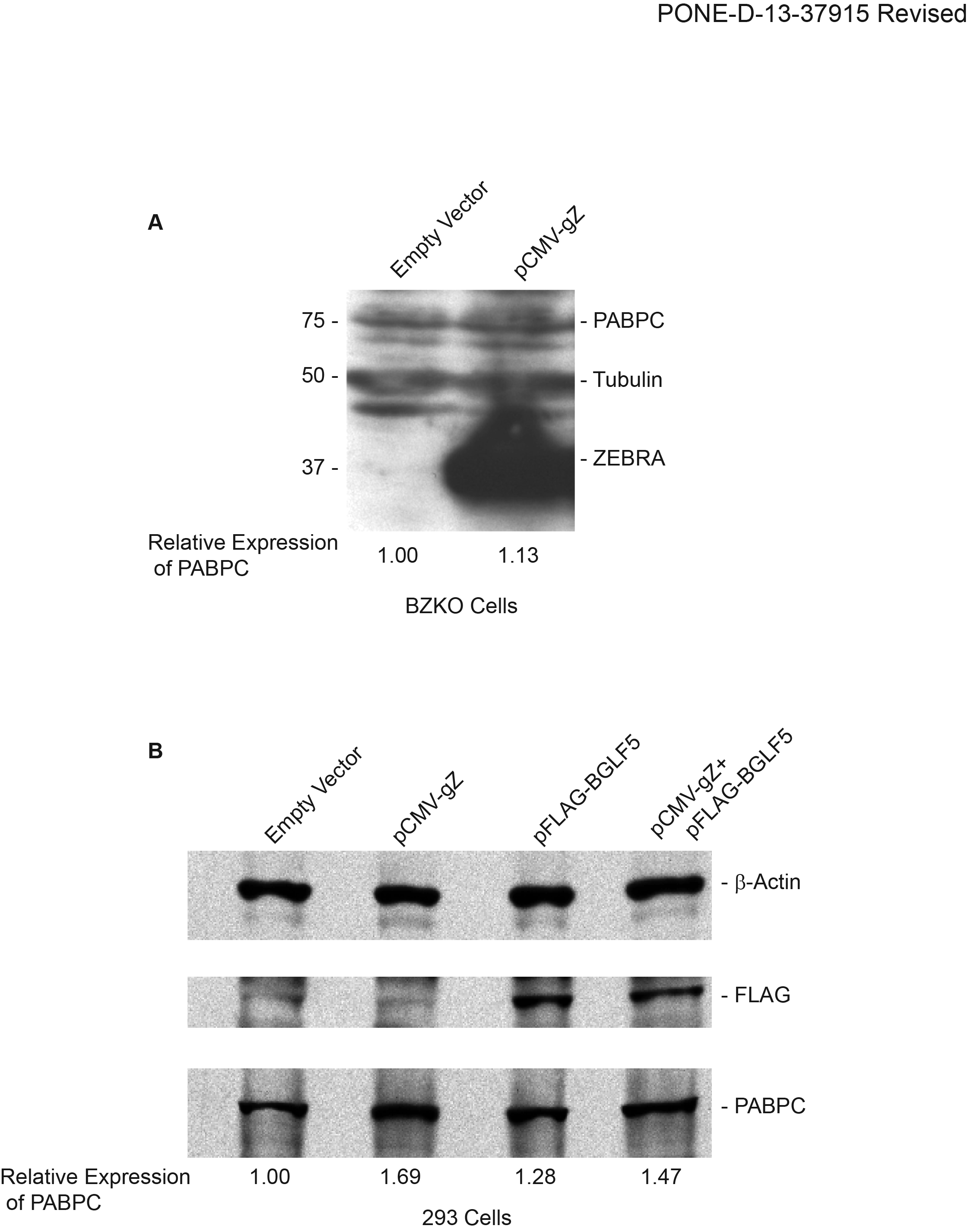

Supplement: Figure S2 — Levels of PABPC during induction of the lytic phase, and during expression of ZEBRA and BGLF5. (A) BZKO cells were transfected with vector (pHD1013) or pCMV-gZ expressing wild type ZEBRA. Cell extracts were prepared 48 h after transfection. Immunoblots were probed with antibodies to ZEBRA, PABPC and tubulin. (B) 293 cells were transfected with vector, ZEBRA or FLAG-BGLF5. Cell extracts were prepared 43 h after transfection. Immunoblots were probed with antibodies to FLAG, PABPC and β-actin. (TIF) [file pone.0092593.s002.tif]

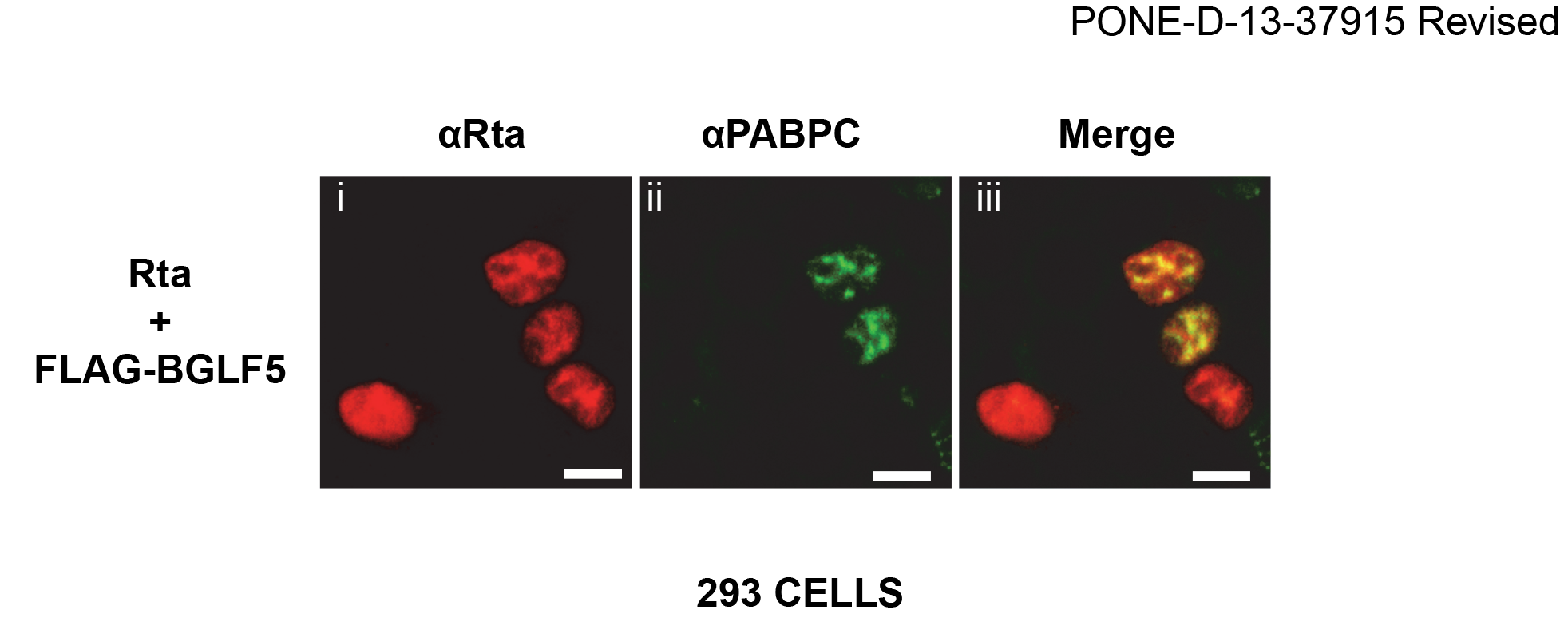

Supplement: Figure S3 — Rta does not redistribute intranuclear PABPC. 293 cells were transfected with Rta and FLAG-BGLF5. Cells were fixed and stained with antibodies specific for PABPC and Rta, and fluorescent secondary antibodies. Reference bar in each panel equals 10 μM in length. (TIF) [file pone.0092593.s003.tif]

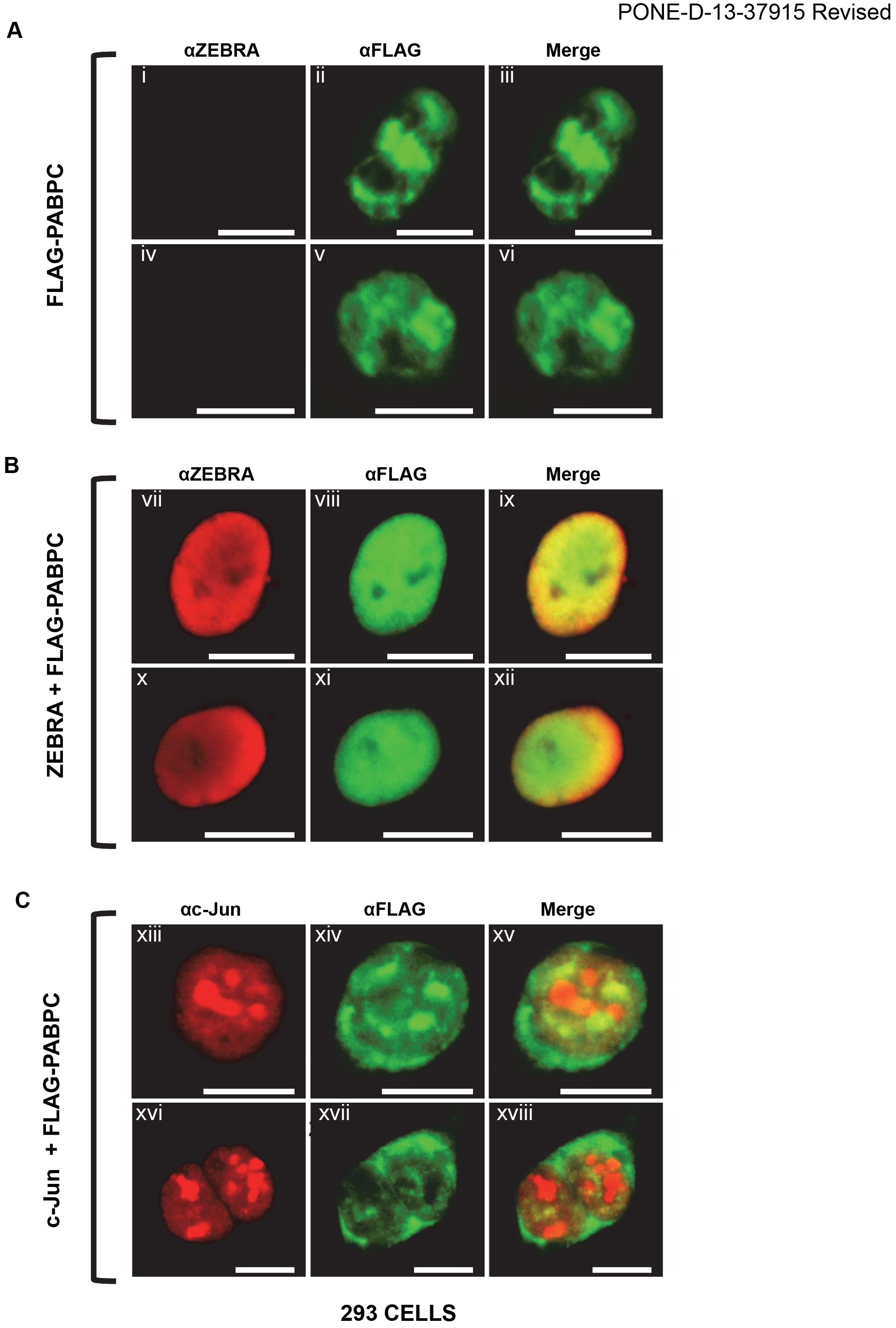

Supplement: Figure S4 — ZEBRA but not c-Jun relocalizes FLAG-PABPC. 293 cells were co-transfected with: (A) FLAG-PABPC, (B) ZEBRA and FLAG-PABPC, (C) c-Jun and FLAG-PABPC. Cells were fixed and stained with antibodies specific for ZEBRA, FLAG, and c-Jun, and fluorophore-conjugated secondary antibodies. Each of the following sets of panels depicts the same field of view: [i-iii], [iv-vi], [vii-ix], [x-xii], [xiii-xv], [xvi-xviii]. Reference bar in each panel equals 10 μM in length. (TIF) [file pone.0092593.s004.tif]

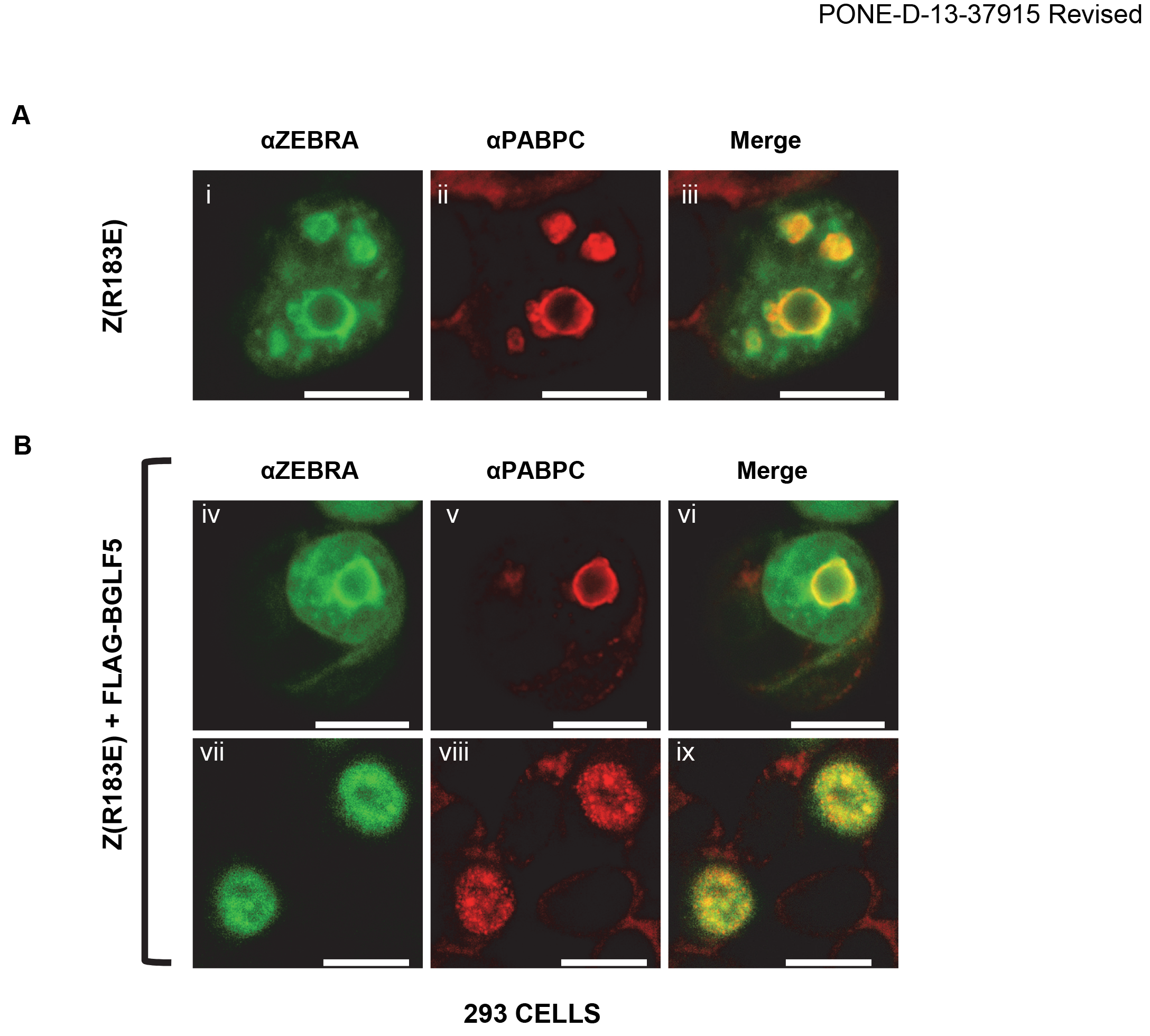

Supplement: Figure S5 — The DNA-binding deficient aggresome-inducing mutant of ZEBRA, Z(R183E), relocalizes PABPC. 293 cells were (A) transfected with Z(R183E) or (B) co-transfected with Z(R183E) and FLAG-BGLF5. Cells were fixed and stained with antibodies specific for ZEBRA and PABPC, and fluorophore-conjugated secondary antibodies. Each of the following sets of panels depicts the same field of view: [i-iii], [iv-vi], [vii-ix]. Reference bar in each panel equals 10 μM in length. (TIF) [file pone.0092593.s005.tif]

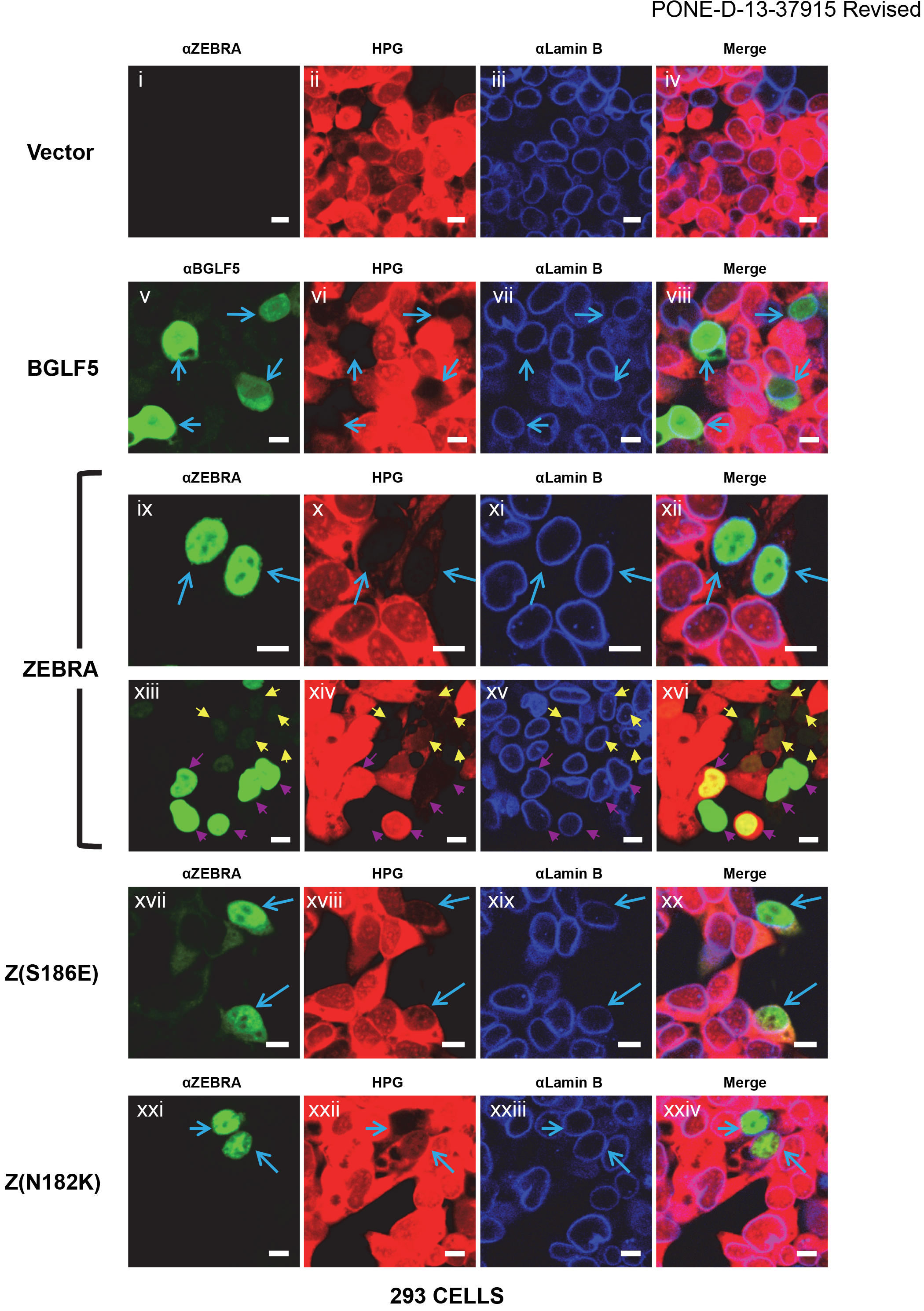

Supplement: Figure S6 — BGLF5 and ZEBRA inhibit endogenous nascent protein synthesis on a global scale; point mutations in the basic region impair ZEBRA's host shutoff activity. 293 cells were transfected with pHD1013, or vectors expressing BGLF5, ZEBRA, Z(N182K), or Z(S186E). Cells were incubated in methionine-free, cysteine-free media containing HPG, then fixed. Using click-chemistry based reagents, incorporated HPG was covalently bound to Alexa Fluor 555. Cells were stained with antibodies specific for ZEBRA and lamin B, and fluorophore-conjugated secondary antibodies. (A) Each of the following sets of panels depicts the same field of view: [i-iv], [v-viii], [ix-xii], [xiii-xvi], [xvii-xx], [xxi-xxiv]. Blue arrows denote cells expressing transfected protein. In panels [xiii-xvi], purple arrows denote cells expressing relatively high levels of ZEBRA, yellow arrows denote cells expressing relatively low levels of ZEBRA. Reference bar in each panel equals 10 μM in length. (TIF) [file pone.0092593.s006.tif]
